# Kaempferol mitigates Endoplasmic Reticulum Stress Induced Cell Death by targeting caspase 3/7

**DOI:** 10.1038/s41598-018-20499-7

**Published:** 2018-02-01

**Authors:** Ahmad Abdullah, Palaniyandi Ravanan

**Affiliations:** Apoptosis and Cell Survival Research Lab, Department of Biosciences, School of Biosciences and Technology, VIT University, Vellore, India

## Abstract

The Endoplasmic Reticulum (ER) plays a fundamental role in executing multiple cellular processes required for normal cellular function. Accumulation of misfolded/unfolded proteins in the ER triggers ER stress which contributes to progression of multiple diseases including neurodegenerative disorders. Recent reports have shown that ER stress inhibition could provide positive response against neuronal injury, ischemia and obesity in *in vivo* models. Our search towards finding an ER stress inhibitor has led to the functional discovery of kaempferol, a phytoestrogen possessing ER stress inhibitory activity in cultured mammalian cells. We have shown that kaempferol pre-incubation significantly inhibits the expression of *GRP78* (a chaperone) and *CHOP* (ER stress associated pro-apoptotic transcription factor) under stressed condition. Also, our investigation in the inhibitory specificity of kaempferol has revealed that it inhibits cell death induced by diverse stimuli. Further study on exploring the molecular mechanism implied that kaempferol renders protection by targeting caspases. Both the *in silico* docking and *in vitro* assay using recombinant caspase-3 enzyme confirmed the binding of kaempferol to caspases, through an allosteric mode of competitive inhibition. Altogether, we have demonstrated the ability of kaempferol to alleviate ER stress in *in vitro* model.

## Introduction

Apoptosis is a quintessential signaling pathway which occurs physiologically during development and maintains normal cellular homeostasis. In contrast, its dysregulation under pathological conditions ranges from cancer resulting due cells evading apoptosis to disorders involving extensive cell loss^1^. Imbalance in cellular proteostasis due to accumulation of misfolded or unfolded proteins triggers signaling pathways in ER called UPR. When ER overwhelms with protein load, GRP78 (78 kDa glucose-regulated protein) dissociates from and activates ER transmembrane proteins: PERK (RNA-like endoplasmic reticulum kinase), ATF6 (Activating transcription factor 6) and IRE1α (Inositol requiring enzyme 1 alpha). UPR primarily fires pro-survival signal transduction pathways aiming to restore homeostasis by decreasing the protein load in ER simultaneously enhancing the machinery for protein folding and degradation. However if the attempt fails, the UPR triggers cell death via apoptosis^2,3^.

Prolonged activation of ER stress contributes to the development and progression of several pathological conditions. These include neurodegenerative diseases, stroke, cardiovascular diseases, obesity, diabetes, cancer, immune disorders, atherosclerosis and liver diseases^4,5^. There are increasing *in vivo* experimental evidences suggesting that alleviating ER stress demonstrates therapeutic potential. Significant improvement have been observed after administration of ER stress inhibitors in *in vivo* models of neuronal injury^6,7^, cardiovascular disease^8^, hypertensive chronic kidney disease^9^, LPS induced lung inflammation^10^, renal ischemia reperfusion injury^11,12^, post traumatic brain injury^13,14^, spinal cord injury^15^ and sleep apnea^16^.

Identification of small molecule inhibitors that can target UPR machinery and reduce ER stress has drawn considerable attention. In the past, several compounds potentially modulating ER stress have been reported. For example, 4-PBA and TUDCA (chemical chaperones)^17^, salubrinal (inhibitor of dephosphorylation of eIF2α)^18^, valproate (inducer of chaperones without evoking the UPR)^19^ and benzodiazepinones (modulators of ASK1)^20^ have been extensively studied.

Many of the current therapeutic drugs marketed have their discovery rooted in traditional medicine as they are either unaltered natural products or their modified synthetic analogues with improved efficiencies. The very same reason has intrigued researchers to screen herbal plants, isolate, identify and evaluate their bioactive compounds as leads for potential drugs. Tailoring the natural products to enhance their potency, availability, selectivity and various other properties have introduced several clinically therapeutic drugs^21–24^. Liu *et al*., have described several naturally occurring saponins, alkaloids and polyphenols that regulate ER stress induced cell death via apoptosis and autophagy^25^. Alternatively, flavonoids are natural antioxidants that have been reported to exert neuroprotective functions in several cell lines and *in vivo* models^26,27^.

In the present study, we performed a small scale screening of selected bioactive compounds and identified a flavonoid, kaempferol^28^, possessing ER stress inhibitory activity *in vitro*. We are the first to report that kaempferol reversed the effects of Brefeldin A (BFA) which is a classic ER stress inducer. Further study on elucidating the working mechanism of kaempferol has revealed that it inhibits BFA induced apoptosis by inhibiting the activity of caspases and maintains the expression of cIAPs in cultured mammalian cells.

## Results

### Kaempferol attenuated ER stress induced cell death in multiple cell lines

**Figure 1:**
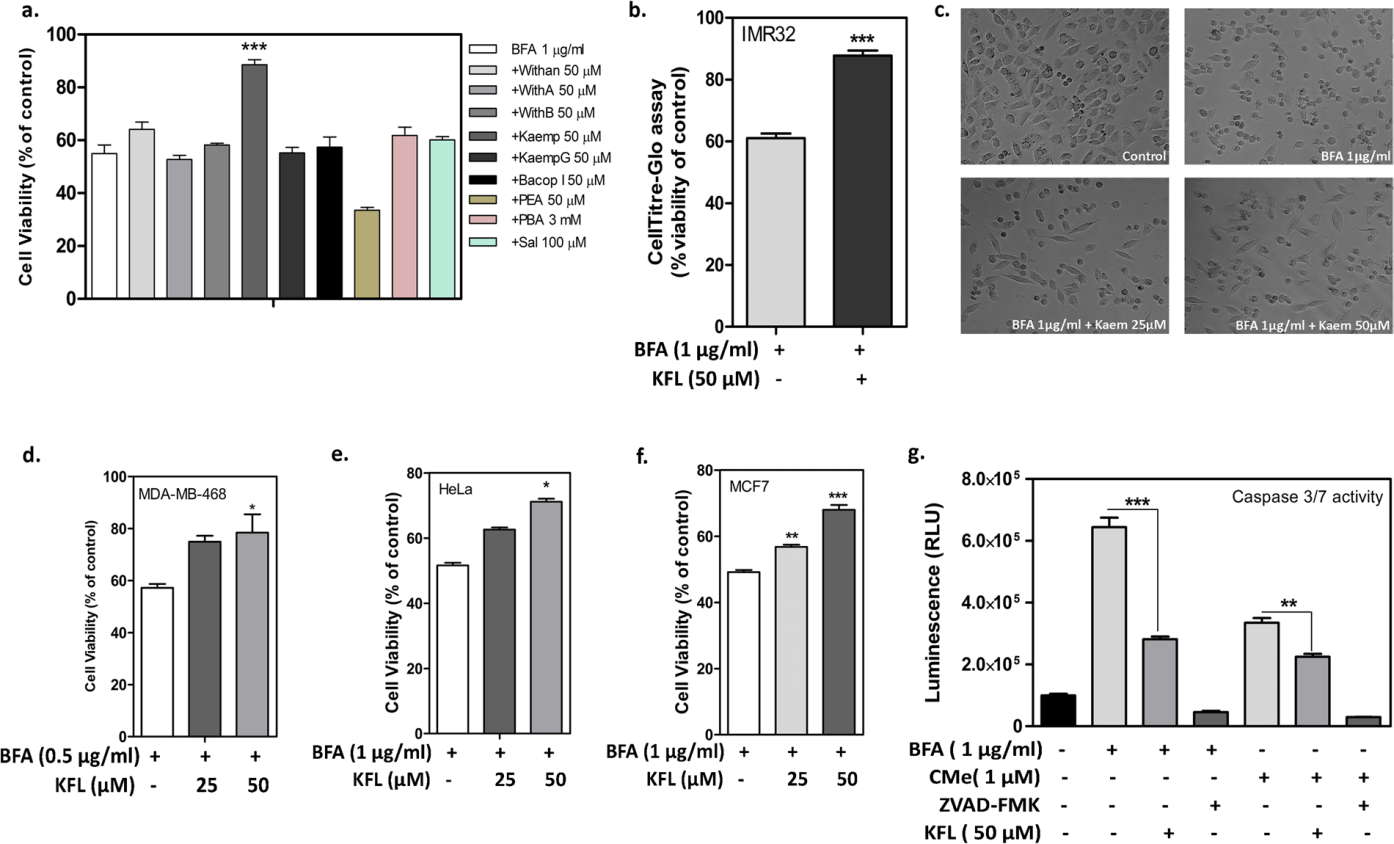
Kaempferol rescues cells against BFA induced cell death. (**a**) Screening for cytoprotective compounds (50 µM) against BFA (1 µg/ml) induced ER stress in IMR32 cell line. Cells were plated and pre-incubated with the test compounds (90 min) and treated with ER stress inducer BFA, incubated for 24 h. Cell viability was estimated by MTT assay. BFA: Brefeldin A; Withan: Withanone; With A: Withanaloide A; With B: Withanaloide B; kaemp: kaempferol; kaempG: Kaempfeol-3-O-robinbioside-7-O-glucoside; Bacop I: Bacopaside I; PEA: Palmitoylethanolamide; PBA: Phenyl Butyric acid; Sal: Salubrinal. (**b**) Confirmation of cytoprotective activity of kaempferol by measuring cellular ATP level using CellTitre Glo reagent. (**c**) Phase contrast images of IMR32 cells after BFA treatment in presence or absence of kaempferol pre-incubation. (**d–f**) Cytoprotective activity of kaempferol against BFA induced cell death in multiple cell lines with incubation time of 24 hours. Cell viability was assessed using MTT assay. (**g**) Inhibition of caspase 3/7 activation by kaempferol against BFA and CDDO-Me induced caspase 3/7 activation in IMR32 cells. Data represents mean ± SEM of experiment performed in triplicate (n = 3). Note: *Represents the significance between cell death inducer alone treated condition compared to kaempferol pre-treated condition, at p ≤ 0.05 (one way ANOVA).

Because ER stress induced cell death is associated with multiple neurodegenerative diseases, we focused our small scale screening of phytocompounds for the ER stress inhibitory activity on human neuroblastoma cell line IMR32. The selection of phytocompounds was based on the plant extracts which are used in traditional Indian medicine for the treatment of neurological disorders. To screen for ER stress inhibitors, IMR32 cells were pre-incubated with the test compounds for 90 minutes and later treated with ER stress inducer BFA (Golgi disruptor-1 µg/ml) to trigger cell death. All the test compounds were screened at a single concentration of 50 µM and MTT assay was performed to assess the percentage of cell survival. Salubrinal and PBA, the known ER stress inhibitors served as positive controls. Of the screened compounds, only kaempferol displayed significant protection against BFA induced cell death. Surprisingly, salubrinal and PBA failed to inhibit cell death induced by BFA in IMR32 cells (Fig. 1a). The structures of screened compounds have been shown in Supplementary Figure 1. Besides BFA, kaempferol also inhibited ER stress induced cell death stimulated by CDDO-Me (2-cyano-3,12-dioxo-oleana-1,9(11)-dien-28-oic acid methyl ester)^29^ (Supplementary Figure 2a). The cytoprotective activity of kaempferol was confirmed by an orthogonal assay that measures the ATP level (CellTiter-Glo luminescent cell viability assay, Promega, India) and trypan blue dye exclusion assay as a surrogate for the cell viability (Fig. 1b and Supplementary Figure 4a). Furthermore, typical characteristics like cell shrinkage and cell detachment was observed in BFA treated cells. However, considerably large number of kaempferol pretreated cells displayed healthy cellular morphology compared to BFA treated cells (Fig. 1c). Because kaempferol exhibited cytotoxicity at higher concentrations, we have set 50 µM as the maximum concentration for further analysis (Supplementary Figure 2b). Furthermore, kaempferol exhibited protection against multiple ER stress inducing stimuli in other cancer cell lines (MDA-MB-468, HeLa, MCF-7 and Neuro2A) as well (Fig. 1d,e and f and Supplementary Figure 2c–f). Reimertz *et al*., has demonstrated that ER stress induced cell death is caspase dependent in neuronal cells^30^; hence we investigated the effect of ER stress on the activity of caspase 3/7 using commercially available Glo-caspase 3/7 assay kit (Promega, India) in IMR32 cells. Our result indicated that, both BFA and CDDO-Me induced the caspase3/7 activity and kaempferol reduced the activity into half (Fig. 1g). We used ZVAD-FMK, a pan caspase inhibitor as a positive control. Collectively, these preliminary results showed that kaempferol attenuates cell death induced by various ER stress inducers in multiple cell lines perhaps due to inhibition of caspase 3/7 activity.

Previous studies have demonstrated that kaempferol is a phytoestrogen (Estrogen receptor β agonist)^31^. Therefore, we tested whether PHTTP (a selective ER β antagonist) has any effect on protection yielded by kaempferol. As shown, CellTiter-Glo luminescent cell viability assay (Supplementary Figure 3a) as well as caspase 3/7 activity assay (Supplementary Figure 3b) results revealed that the ER β antagonist couldn’t reverse the protection exerted by kaempferol, suggesting that kaempferol did not require active estrogen receptor signaling pathway to inhibit BFA induced cell death. Simultaneously, we tested if another ER β agonist DPN (Diarylpropionitrile) and the estrogen (17β-estradiol) reduces BFA induced caspase 3/7 activity in IMR32 cells. As shown in Supplementary Figure 3c, Estrogen receptor modulators failed to inhibit caspase 3/7 activity (Supplementary Figure 3c). These findings suggest that kaempferol inhibits ER stress induced apoptosis in multiple cell lines; the anti-apoptotic effect of kaempferol is mediated via inhibition of caspase 3/7 activity and it is estrogen receptor signaling independent.

### Mapping the UPR/apoptotic pathways regulated by kaempferol

**Figure 2:**
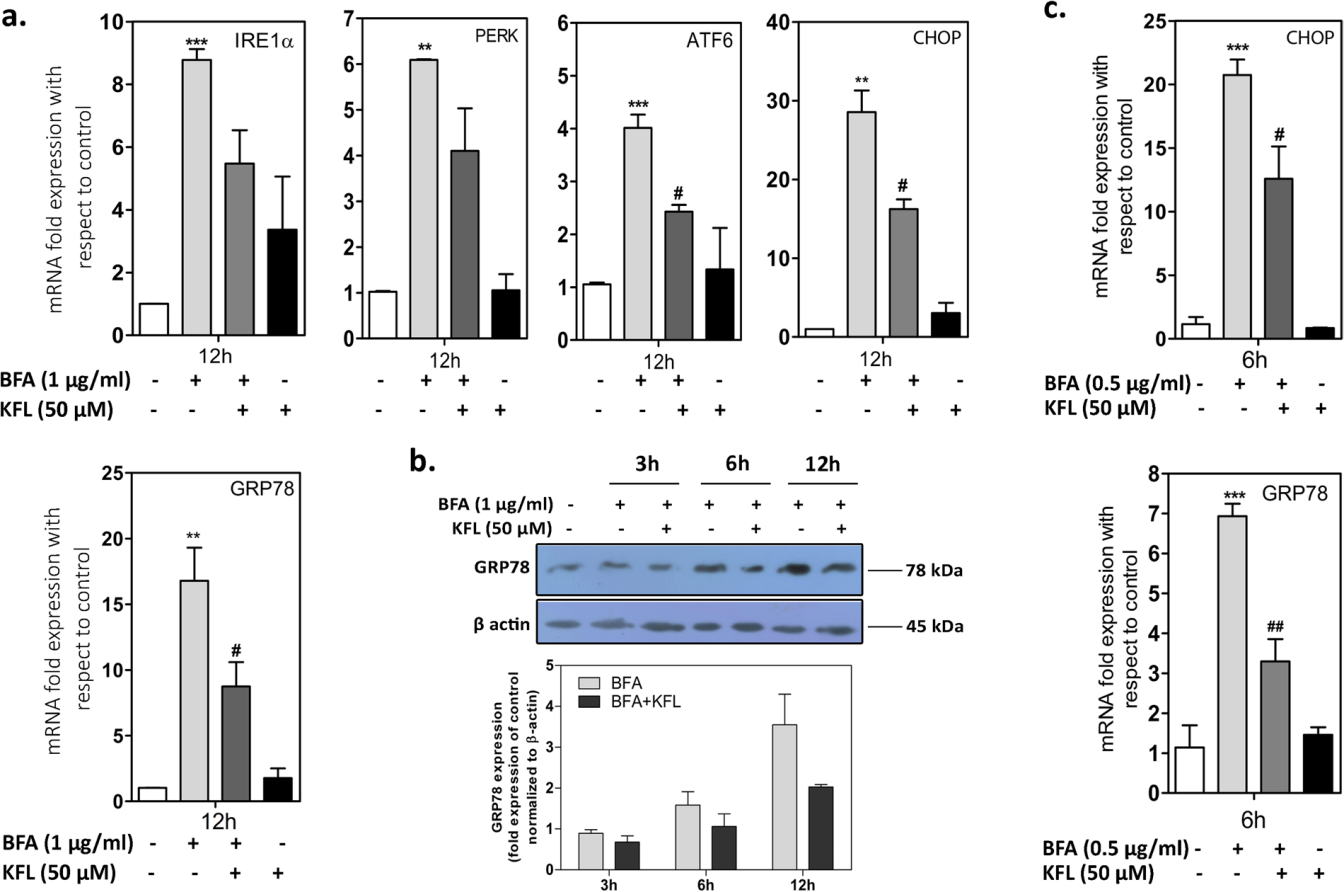
Kaempferol inhibits UPR signaling (**a**) IMR32 cells were cultured with BFA in presence or absence of kaempferol pre-incubation. Q-RTPCR was performed to measure the expression of genes (IRE1α, PERK, ATF6, GRP78 and CHOP) involved in UPR signaling. (**b**) Immunoblot representing the reduction in the expression of GRP78 in BFA treated conditions upon pretreatment with kaempferol. Densitometry analysis of GRP78 was performed. Data represents the average ± SEM of two independent experiments (n = 2). (**c**) MDA-MB-468 cells were cultured with BFA in presence or absence of kaempferol pre-incubation. Q-RTPCR was performed to measure the expression of GRP78 and CHOP. Data represented as average ± SEM of two independent experiments performed in duplicate. *Represents the significant increase in expression of BFA treated cells with respect to control cells; ^#^represents the reduction in gene expression in kaempferol pre-treated cells when compared to BFA alone treated cells (p ≤ 0.05; one way ANOVA).

ER stress triggers UPR signaling cascade which is initiated by GRP78, a master regulator in association with IRE1α, PERK and ATF6 (reviewed in Chaudhari *et al*., 2014)^32^. We performed real time quantitative RT-PCR (q-RT-PCR) and immunoblotting to study the impact of kaempferol in the regulation of UPR signal transduction pathways. As shown in Fig. 2 and Supplementary Figure 4b, BFA induced the expression of GRP78, IRE1α, PERK and ATF6; however, the cells pre-incubated with kaempferol showed significant reduced expression level of GRP78 and ATF6 mRNA, while IRE1α and PERK expression was not significantly altered in IMR32 cells (Fig. 2a). GRP78 induction is considered as a marker for ER stress, showed reduced expression in protein level in kaempferol pretreated cells (Fig. 2b). Recent study published by Guo *et al*. showed kaempferol induces ER stress, thereby inducing cell death in HepG2 hepatocellular carcinoma cells^33^. In our study, although higher concentration of kaempferol (100 µM) exhibited cytotoxicity, it failed to induce Grp78 expression in IMR32 neuroblastoma cells and reduced BFA induced expression of Grp78 levels (Supplementary Figure 4c,d). Therefore the activation of ER stress by kaempferol reported in other tumor cell lines might be a cell line specific and concentration dependent event but not a general mode of action by kaempferol. Moreover, the authors have mentioned the apoptotic activity as concentration and time dependent in HepG2 cells. Bax and SIRT3 activation, p53 activation and ROS generated oxidative stress are other mode of kaempferol induced cell death reported in various tumour cell lines^34–36^. All three UPR signaling events finally converge into the activation of CHOP (C/EBP homologous protein), a pro-apoptotic transcription factor that induces apoptosis by modulating the expression of several genes including Death Receptor-DR5 (a known CHOP target gene), pro-apoptotic gene Bim (Bcl-2 interacting mediator of cell death) and anti-apoptotic gene Bcl-2 (B-Cell Lymphoma-2). In this aspect, CHOP has been shown to induce the expression of Bim while reducing the expression of Bcl-2. As shown in Fig. 2a and c, pre-treatment with kaempferol considerably attenuated BFA induced upregulation of CHOP mRNA levels in two different cell lines (IMR32 and MDA-MB-468).

**Figure 3:**
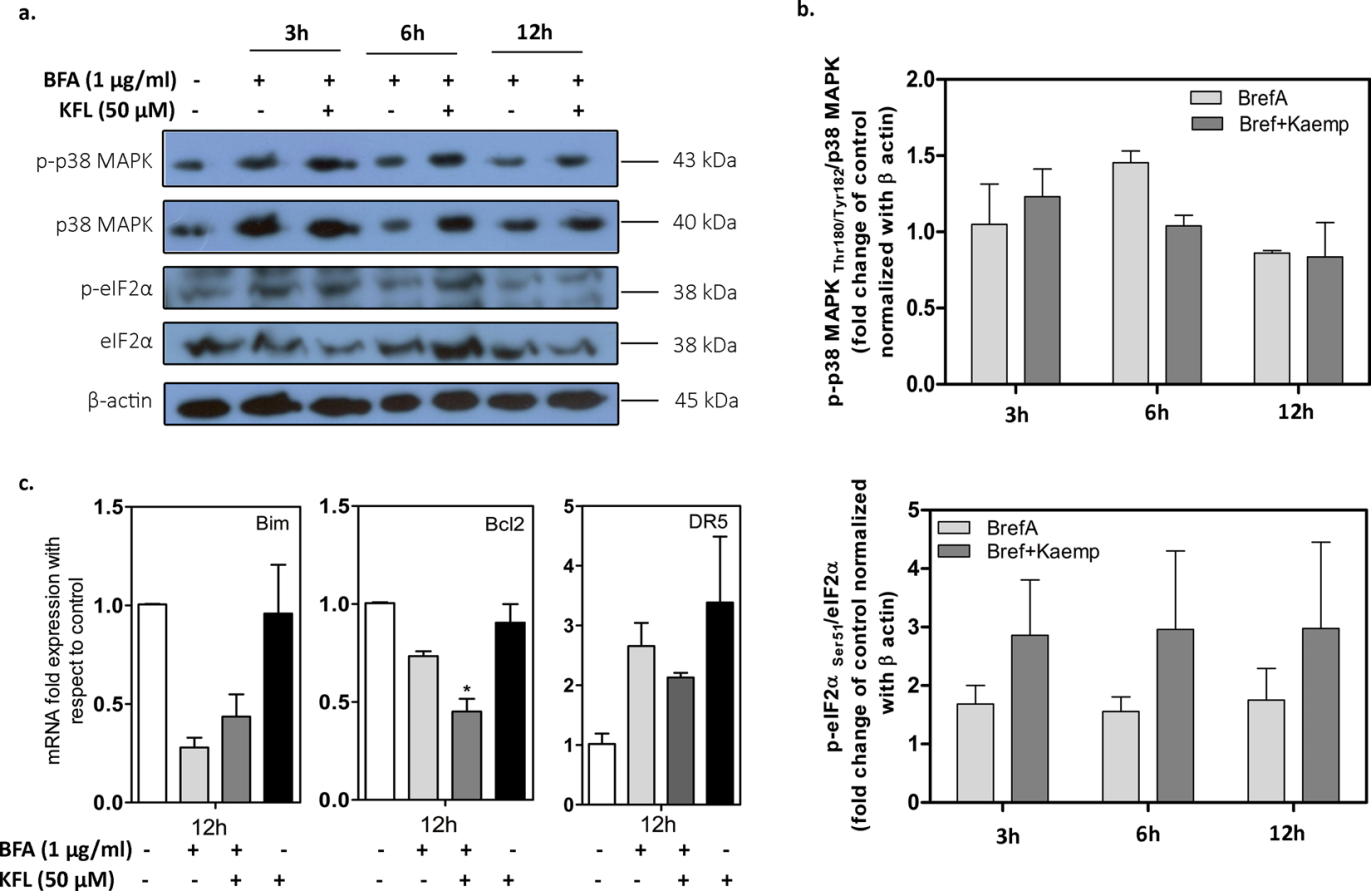
Kaempferol regulates ER stress induced apoptotic signaling. (**a**) IMR32 cells were treated with BFA in presence or absence of kaempferol pre—incubation for 3 h, 6 h and 12 h. Lysates were prepared, normalized for total protein content, and analyzed by immunoblotting using antibodies specific for p-p38-MAPK, p38-MAPK, p-eIF2α. (**b**) Densitometry analysis of ratio of phospho to non-phosphorylated levels of p38 MAPK and eIF2α. Data represents ratios of the average ± SEM of two independent experiments (n = 2). (**c**) Regulation of Bim, Bcl2 and DR5 gene expression upon BFA treatment in presence and absence of Kaempferol pre-treatment. Data represented as mean ± SEM of two independent experiments performed in duplicate. ^*^Represents the significance with respect to control cells (p ≤ 0.05 (one way ANOVA).

Depending on the intensity and duration of ER stress, IRE1α activates various stress signals including JNKs (c-Jun N-terminal kinase) and P38 MAPK (Mitogen activated protein kinase) which can eventually lead to cell death. To measure the p38MAPK activation, immunoblotting was performed. As shown, BFA induced both p38MAPK and phosphorylated p38MAPK while, kaempferol reduced the activation partially at 6 h but we did not observe any change in the levels of phospho-p38 at 3 h and 12 h (Fig. 3a,b). PERK activation initiates a translational arrest by phosphorylating the eIF2α and thereby decreasing the ER protein load. Treatment of cells with kaempferol increased the level of phospho-eIF2 alpha, suggesting that this mechanism could contribute to the protection exerted by kaempferol against BFA induced ER stress.

In IMR32 cells, BFA up regulated DR5 mRNA expression. This finding has been previously reported in various cell types using different ER stress inducers^29,37^. Interestingly, similar to BFA, kaempferol treatment also induced the expression of DR5. In spite of significant upregulation of DR5 level in kaempferol alone treated cells, we did not observe activation of caspase 3/7 (Supplementary Figure 3c) suggesting that DR5 upregulation did not have any effect on caspase 3/7 activation and cell death in IMR32 cells. Pertaining to Bcl-2 gene expression, BFA treatment alone reduced Bcl-2 expression while pre-incubation with kaempferol decreased the expression further. Notably, kaempferol alone did not change the expression of Bcl-2 and Bim (Fig. 3c). Collectively these results indicate that kaempferol decreases the mRNA expression of certain BFA regulated genes involved in ER stress and cell death with simultaneous inhibition of BFA activated stress kinases.

### Inhibition of caspase 3/7 activity by kaempferol maintains the expression of IAPs in IMR32 cells

**Figure 4:**
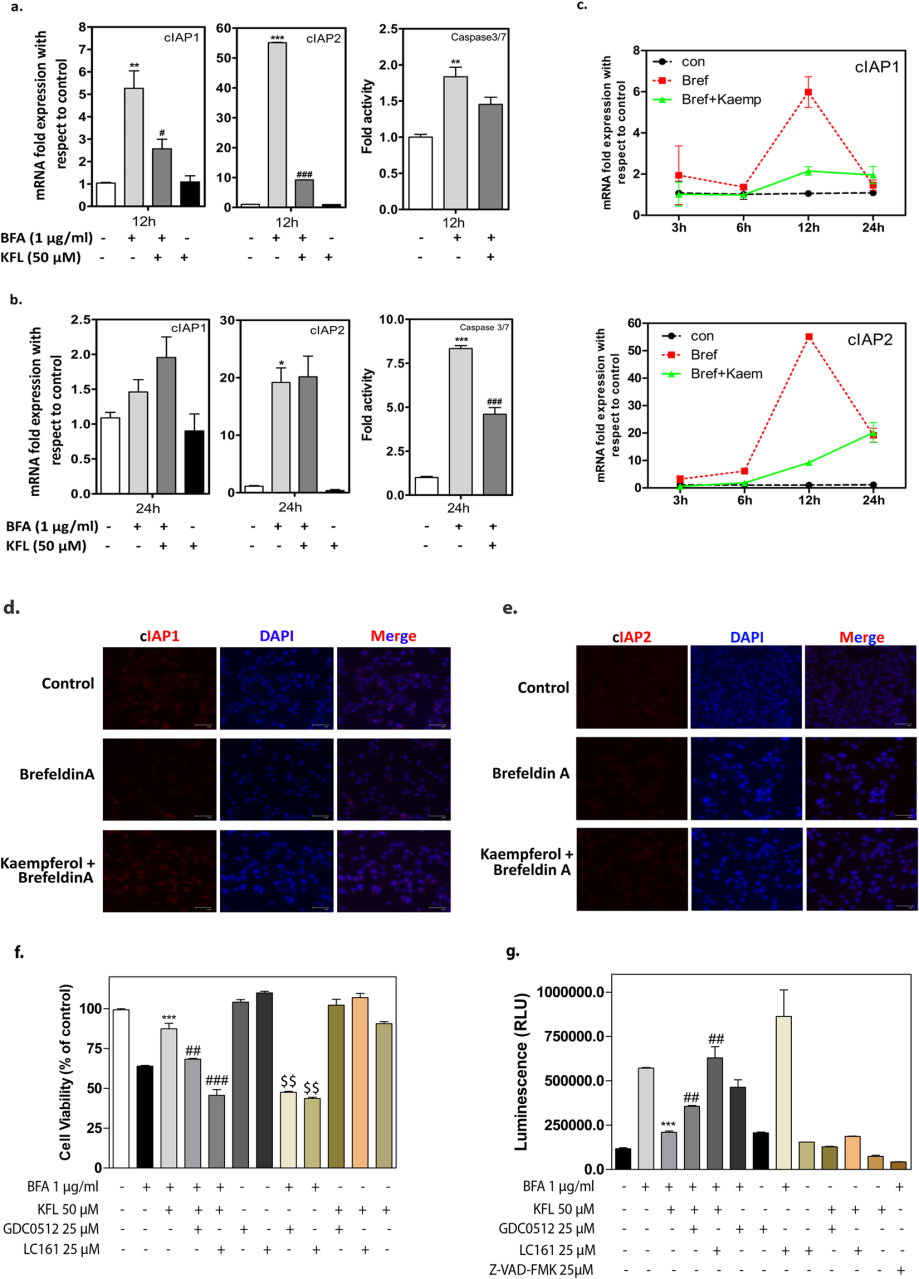
Kaempferol maintains cIAPs and inhibits caspase 3/7 activation. (**a**,**b**) Expression of cIAP1 and cIAP2 mRNA level was measured by q-RTPCR. ER stress was induced in IMR32 cells with BFA in the presence and absence of kaempferol pre-treatment. Expression of cIAPs level was compared to caspase 3/7 activation in 12 h and 24 h of ER stress induction. Data represented as average ± SEM of two independent experiments performed in duplicate. ^*^Represents the comparison of BFA treated condition to control cells; ^#^represents the significant difference between BFA treated cells to BFA + kaempferol treated cells at p ≤ 0.05. (**c**) Regulation cIAPs mRNA level at increasing treatment hours in IMR32 cells cultured with BFA with/without kaempferol pre-treatment. Data represented as average ± SEM of two independent experiments performed in duplicate. (**d,e**) Protein levels of cIAP1 and cIAP2 in IMR32 cells after 24 hours of treatment with BFA and kaempferol pre-treated conditions by immunocytochemistry (Scale bar: 67 µm). (**f**) MTT assay showing the reversal of cell death protection by SMAC mimetic compounds co-treatment with KFL + BFA treated cells after 24 hours of incubation. (**g**) SMAC mimetic increases the caspase 3/7 activity in IMR32 cells upon treated with BFA alone and in BFA + KFL combination. Data represented as average ±SEM of two independent experiments performed in triplicate. * and ^$^ Represents significance at p < 0.05 with respect to BFA treated cells and ^#^Represents significance at p < 0.05 with respect to BFA + KFL treated cells (One-way ANOVA).

The family of cysteine proteases named caspases is critical mediators of apoptosis. Caspases are mainly divided into initiator caspases (caspase-8, -9 and -12) and executioner caspases (caspase-3, -6, and -7). In ER stress induced cell death, caspase-12 has been demonstrated as the initiator caspase while procaspase-9 has been shown as substrate for caspase-12^38^. Caspases activation can be further regulated by endogenous inhibitors of apoptosis proteins (IAPs) and SMAC/Diablo. IAPs are conserved throughout animal evolution and several IAP-family proteins have been shown to directly bind and inhibit the activity of caspases, thereby suppressing apoptosis. Evidences support that increased cIAPs (cellular inhibitor of apoptosis protein) levels protect cells against apoptotic stimuli^39^. Hence, we sought to investigate if kaempferol regulated IAPs as a self-defense mechanism against ER stress induced cell death. We studied the mRNA expression profile of cIAP1, cIAP2, XIAP (X-linked inhibitor of apoptosis protein) and survivin after treatment with BFA in presence and absence of kaempferol. Our results showed that BFA treatment induced cIAP1 and cIAP2 mRNA level to 5 and 55 folds respectively at 12 hours which was reduced to 3 and 10 folds respectively in kaempferol pre-treated cells. Similarly, BFA increased the levels of cIAP1 and cIAP2 to 1.5 and 20 folds respectively at 24 hours, but for kaempferol pre-treated cells, cIAPs levels remained relatively stationary (Fig. 4a–c). We analysed the protein level of cIAPs using immunocytochemistry and results showed that there is a reduction in cIAPs protein level in BFA treated cells despite a high induction in mRNA level. However, in kaempferol pre-treated condition the cIAPs expression was not altered in both mRNA level and protein level between 12 and 24 hours after the induction of ER stress (Fig. 4d,e and Supplementary Figure 6). Analysis of active caspase 3/7 showed that BFA treatment increased the caspase 3/7 activity in 12 hours to 2 folds, wherein we observed simultaneous hike in cIAP1 (~6 fold) and cIAP2 (~50 fold) levels. But, after 24 hours of BFA treatment we observed an elevation in caspase 3/7 activity to 8 folds with a fall in cIAP1 (~1.5 fold) and cIAP2 (~20 fold) mRNA expression. As shown in Fig. 4c BFA induced expression of cIAPs mRNA could be an adaptive mechanism to overcome ER stress induced apoptosis. In kaempferol pre-treated cells, the activity of caspase 3/7 by BFA was inhibited significantly to the level of 1.4 and 4 fold at 12 and 24 hours respectively (Fig. 4a,b). Both the cIAPs expression level was maintained in kaempferol pre-treated cells; this suggests the inhibitory action of kaempferol on caspases, which otherwise had to be done by increase in the levels of cIAPs expression^40^. We also observed that kaempferol treatment alone did not induce caspase 3/7 activation despite kaempferol known to cause cell death at higher concentration (Supplementary Figure 3c). Similarly, kaempferol did not alter other IAPs like XIAP and survivin gene expression in IMR32 cells (Supplementary Figure 5), which corresponds to the study published by *Harlin et al*., on an increase of cIAPs expression in XIAP deficient mice^41^. Taken together, these results suggest that kaempferol inhibits caspase 3/7 activity and maintains the levels of cIAPs in IMR32 cells.

### IAPs antagonist (SMAC mimetics) reverses the protection by kaempferol

SMAC mimetics are small molecules which inhibit the activity of IAPs by directly binding to the shallow groove on the surface of IAP’s BIR domains or indirectly inhibits by inducing auto-ubiquitination followed by proteasomal degradation of IAPs^42^. Using commercially available SMAC mimetic compounds (GDC0152, LC 161, MedChem Express) we tested if these compounds reverse the ER stress inhibitory activity of kaempferol. IMR32 cells were pre-incubated with SMAC mimetics and kaempferol for 90 minutes each and ER stress was induced with BFA. As shown in Fig. 4f,g, viability assay and caspase 3/7 activity assay confirmed that both the SMAC mimetics could reverse the kaempferol induced ER stress inhibition suggesting that IAPs are partly involved in protection mediated by kaempferol. Also, SMAC mimetics alone treated cells did not exhibit cell death as these compounds often require addition of death ligands such as TNFα or TRAIL for the induction of cell death^43^. As expected, BFA induced the cell death as well as caspase 3/7 activity in IMR32 cells while kaempferol pretreatment inhibits both. We observed a significant increase of cell death in BFA + LC161 condition when compared to the BFA alone suggesting that IAP inhibition increases BFA induced caspase activity. Additionally, we observed that kaempferol slightly reduced BFA + LC161 induced caspase activity. ZVAD-FMK was served as a positive control for the caspase 3/7 inhibition.

### Kaempferol alleviates cell death induced by multiple stimuli through the inhibition of caspases

**Figure 5:**
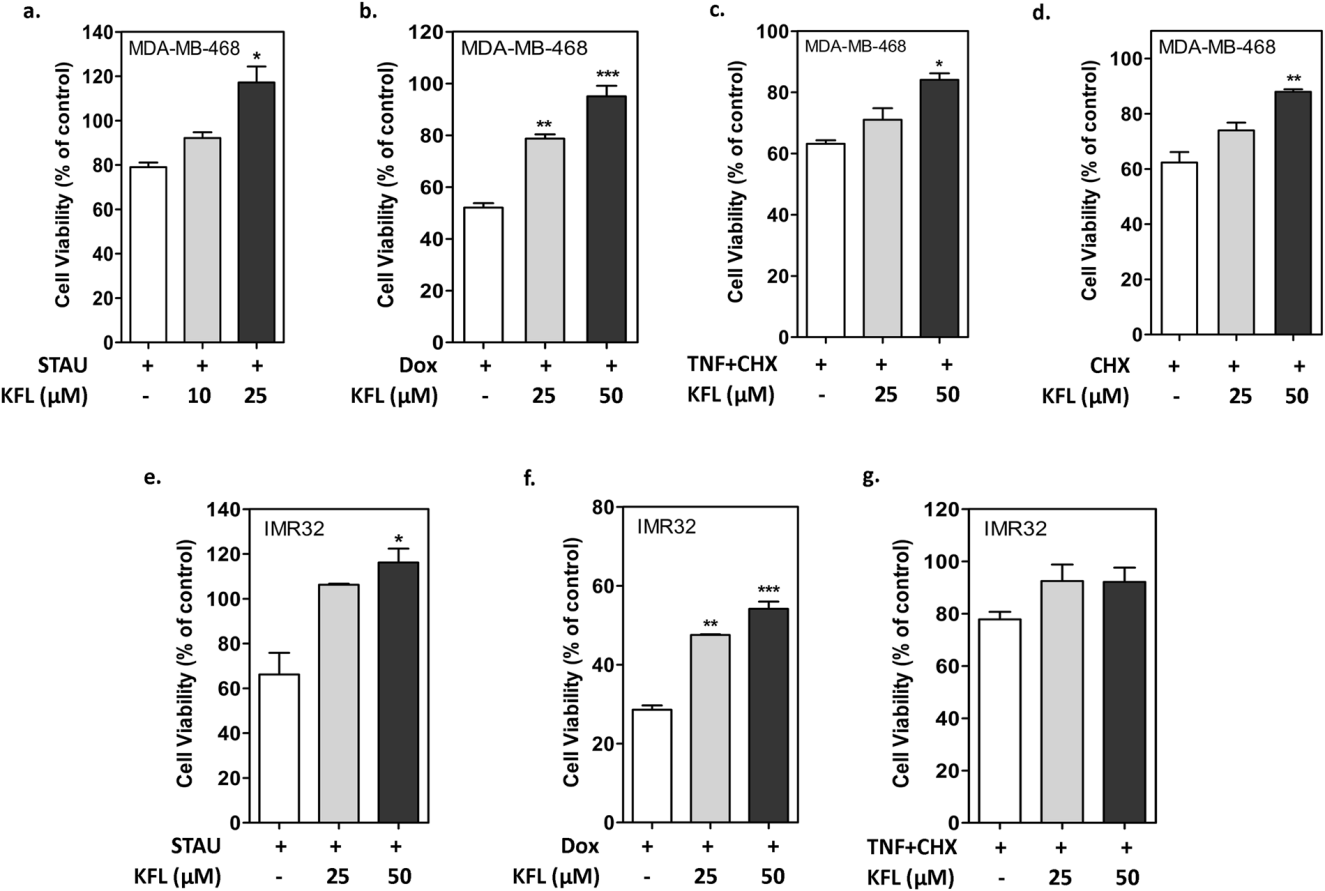
Kaempferol is a general inhibitor of cell death. MDA-MB-468 cells (**a–d**) and IMR32 (**e**–**g**) were treated with staurosporine (100 nM); doxorubicin (1 µg/ml); TNF-α (1 ng/ml) + cycloheximide (0.5 µg/ml) and cycloheximide (25 µg/ml) alone or after pre-incubation with kaempferol. MTT assay was performed after 24 hours to quantitate percentage of cell death. Data represented as mean ± SEM of at least two independent experiments performed in triplicate. ^*^Represents the significance between cell death inducer alone treated condition compared to kaempferol pre-treated condition, at p ≤ 0.05 (one way ANOVA).

Considering the fact that caspases are involved in cell death induced by various stimuli, we questioned the specificity of kaempferol in inhibiting cell death/apoptosis. To determine, kaempferol’s anti-apoptotic activity is specific to ER stress induced cell death versus general inducers of cell death, we challenged IMR32 and MDA-MB-468 cells to various cell death inducing stimuli, such as TNFα (a cytokine and cell signaling protein)/Cycloheximide (protein synthesis inhibitor), Staurosporine (pan-kinase inhibitor) and Doxorubicin (DNA-intercalating agent). Our results showed that kaempferol inhibits apoptosis induced by multiple stimuli (Fig. 5a–g) confirming that kaempferol may target a key player which is common to most forms of cell death such as caspases.

In contrast to our result, kaempferol has been widely reported to induce cell death in multiple cell lines by inducing the cleavage of caspase zymogens which was shown by western blot. However, the authors failed to test the activity of caspases after the cleavage of caspase-3 which implies that flavonoids may be inhibiting the active form but the cell death is happening possibly via caspase independent mechanism^44–47^. Additionally, induction of cell death in caspase-3^−/−^/caspase-7^−/−^ mouse embryonic fibroblasts (MEF) treated with flavonoids did not show any significant difference in extents of cell death when compared to the wild type MEF cells. These results suggest that, the mechanism of flavonoid induced cytotoxicity is unclear and may involve a caspase independent form of cell death^48^.

### Kaempferol binds to the dimerization interface of caspases

**Figure 6:**
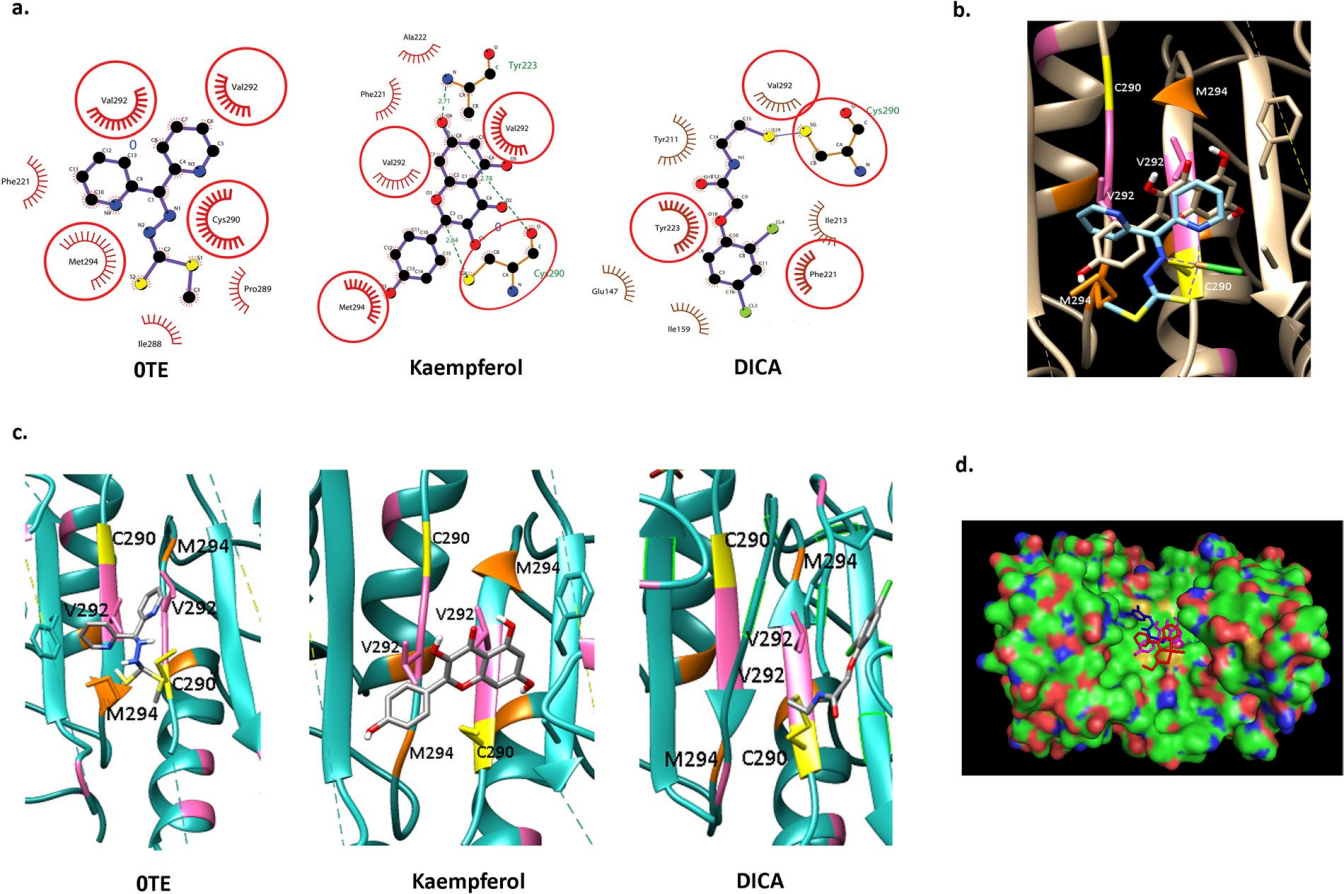
Kaempferol binds to the caspase-7 dimerization site: *In silico* analysis. (**a**) LigPlot^+^ analysis (2D representation) of docked complexes of 0TE and kaempferol compared to crystal structure (PDB ID: 1SHJ) of DICA docked pose with caspase 7 enzyme dimerization site. Red circles represents the amino acid residues common to the binding site of 0TE and DICA, obtained by docking studies with kaempferol. Green dotted lines represents H-bonding (kaempferol) in docked complex and violet solid line (DICA) represents the covalent bonding as represented in the crystal structure. (**b**) Superimposed structures of 0TE and kaempferol docked complexes as represented by UCSF Chimera. (**c**) 3D representation of docked poses of 0TE, kaempferol and DICA using PYMOL (Cysteine ; Valine ; Methionine ) (**d**) Solid Surface representation of caspase-7 dimer with 0TE ; kaempferol ; and DICA ; docked poses superimposed at the dimerization site.

We then employed *in silico* approach (blind docking method) to check if kaempferol efficiently binds to caspase-7 enzyme which shares 53% of sequence identity and 100% (16 out of 16) of the amino acids facing the central core of the enzyme dimer with caspase 3^49^. Both the caspases have same substrate preference as well^50^. Docking with crystal structure of caspase-7 (PDB ID: 4FEA) showed the binding of kaempferol to the dimerization site through H-bonding interaction with the Cys 290 residue (Fig. 6a) which is crucial for the active dimer conformation^49^. The docking studies revealed the interaction of kaempferol in the same binding site as of 0TE a known pan-caspase inhibitor (Fig. 6b). Moreover, we observed the presence of hydrophobic interactions with Val 292 and Met294, which are shown to have a prominent role in binding to the pan caspase inhibitor 0TE^50^. The comparative studies with the docked pose of crystal structure of DICA bound caspase-7 complex (Fig. 6c) also showed that the docked pose of kaempferol signifies a valid mode of caspase inhibition. The solid surface representation of caspase-7 enzyme with docked poses of 0TE, kaempferol and DICA shows the binding of the compounds into the same allosteric site of caspase-7 dimer (Fig. 6d). Docking scores obtained for kaempferol (ΔG = −4.01 kcal/mol) showed significant binding energy as of 0TE (ΔG = −2.83 kcal/mol) re-docked to caspase-7 crystal structure (PDB ID: 4FEA).

We also examined binding of kaempferol to caspase-3 by docking the compound against the crystal structure of caspase-3 with PDB ID: 3DEJ. Since there is no crystal structure of allosteric inhibitor bound to caspase-3 dimerization sites available on PDB, the docked complex was compared to docked complex of 0TE. In both the dockings, there was no interaction found with Cys residue present at the dimerization site. But the LigPlot analysis showed H-bonding for 0TE and kaempferol at Arg 164, Tyr 197 and Glu 124, Arg 164, Thr 140, Tyr 195 of caspase-3 respectively. Moreover, hydrophobic interactions with Val 266, Lys 137, Leu 136, Pro 201 and Gly 125 were also observed (Supplementary Figure 7a–c). Studies performed by *Walters et al*., showed that the mutant V266H caspase-3 causes conformational changes which propagates through the structure and inactivates the enzyme. In the same study, the mutation of Glu 124 and Tyr 197 also showed reduced enzymatic activity. In total the residues Glu 124 and Tyr 197 are involved in the stabilization of Arg 164 residue of the monomer^51^. The surface representation of caspase-3 enzyme structure with superimposed docked poses of 0TE and kaempferol shows that the compounds sharing the same binding pocket at dimerization site, as predicted by docking studies (Supplementary Figure 7d). Thus, the docked complex of 0TE and kaempferol showed interactions with amino acid residues required for the formation of an active caspase-3 dimer thereby may have an active role in the inhibition of enzymatic activity.

Next, docking study was performed on binding of kaempferol to caspase-9 monomer. Our results showed that kaempferol binds to the binding region of BIR3 domain of XIAP protein on caspase-9 monomer (PDB ID: 1NW9) (Supplementary Figure 8a,b). Activation of caspase-9 requires the dimerization of monomers, which was shown to be held inactive by the hetero-dimerization with XIAP-BIR3 domain interacting via Vander Waal’s interaction toLeu384, Leu385, Ala388, and Cys403 residues of caspase-9 monomer^52^. These amino acid residues which are involved in the event of caspase-9 dimerization (for its active state dimer) are being intercepted by the binding of kaempferol (Supplementary Figure 8c). Gln 388 and 381 of caspase-9 forms a hydrogen bond to the hydroxyl moiety of kaempferol, while the 2D interaction analysis with Ligplot^+^ showed the hydrophobic interactions with Leu 384, Ala 388, Met 400, Pro 401 and Cys 403 residues of caspase-9 monomer (Supplementary Figure 8d).

Since kaempferol is a known ER β agonist, we performed the docking analysis for other well-known ER β modulators like 17β-estradiol, DPN and PHTPP (4-[2-Phenyl-5,7-*bis*(trifluoromethyl)pyrazolo[1,5-*a*]pyrimidin-3-yl]phenol). 17β-estradiol showed no favorable interaction with caspase-3 and 7 enzymes in docking studies. DPN and PHTPP interact effectively with caspases-3, 7 & 9 and the results have been tabularized (Supplementary Table 1).

### Inhibitory action of kaempferol against human active caspase-3 enzyme

**Figure 7:**
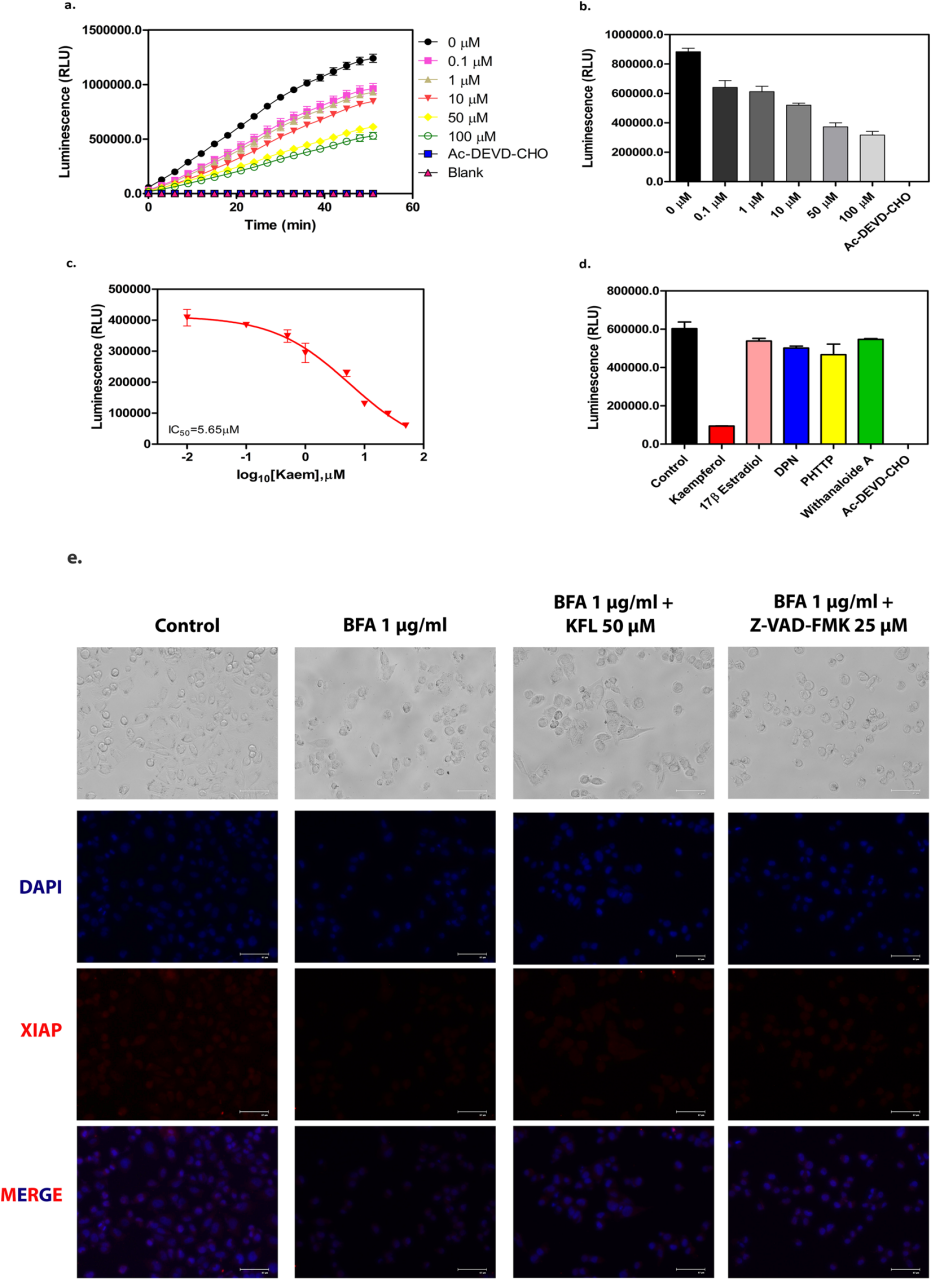
Kaempferol inhibits the activity of caspase-3 enzyme. (**a** and **b**) Caspase-3 inhibition by kaempferol was measured using recombinant caspase-3 (enzyme) and Z-DEVD-aminoluciferin (substrate) with respect to time (**a**) and concentration (**b**). Ac-DEVD-CHO served as a positive control. (**c**) Dose response curve for kaempferol inhibition on caspase-3 enzyme activity. (**d**) Analysis of the inhibitory potential of estrogen receptor modulators on caspase-3 enzyme activity. Withanaloide A served as (−) control and Ac-DEVE-CHO as (+) control. Data represented as mean ± SEM of two independent experiments performed in duplicate. (**e**) Immunocytochemistry for XIAP expression levels in IMR32 cells after 24 hours of incubation in respective conditions (Scale bar: 67 µm).

We subsequently performed *in vitro* assay to study the direct inhibitory activity of kaempferol on caspase-3 using the recombinant active caspase-3 enzyme (Enzo life Sciences) and caspase 3/7 activity assay kit (Promega, India). As shown in Fig. 7a,b the activity of caspase-3 was inhibited by kaempferol in a dose dependent manner and the IC_50_ value was assessed to be 5.65 µM (Fig. 7c). The Michaelis-Menten study for V_max_ and K_m_ showed an increase in K_m_ value with increasing concentration of kaempferol (Table 1), revealed the kaempferol’s competitive mode of inhibition. We used Ac-DEVD CHO, a known peptide inhibitor of caspase-3 as a positive control. Additionally, we tested other ER β modulators for their ability to inhibit caspase-3 activity. Although a high dock score was obtained for these compounds, they failed to reflect in *in vitro* enzymatic assay performed using purified recombinant caspase-3 enzyme. We validated the docking data for DPN, PHTPP and 17β-estradiol also. The results showed that all three compounds were not able to inhibit the caspase-3 activity. However, DPN and PHTPP reduced the caspase-3 activity moderately at very high concentration (150 µM), whereas 17β-estradiol did not inhibit caspase-3 even at 150 µM concentration (Supplementary Figure 9a,e).

**Table 1:** Kinetics parameter of Caspase-3 enzyme activity in presence of Kaempferol.

Kaempferol (µM)	0.0	0.1	1.0	50
V_max_	4.309e + 006	7.536e + 006	1.056e + 007	2.086e + 007
K_m_	120.9	334.2	507.7	1663

Further studies on, whether the inhibition of caspase 3 activity inhibiting the cleavage of caspase-3 downstream targets, we checked the level of XIAP, a target of the caspase-3 enzyme. The studies performed by Walsh JG *et al*., showed preference of caspase-3 enzyme for XIAP over caspase 7^53^, and Hornle *et al*., had shown the cleavage of XIAP by caspase-3 as a prerequisite for its proteasomal degradation^54^, having a predicted cleavage site motif of RSESD-AVSSD, with SNP at Asp242Glu^55^. The results of immunocytochemistry showed the inhibition of XIAP degradation in BFA treated condition in the presence of kaempferol. Although the positive control for caspase-3 inhibitory activity Z-VAD-FMK couldn’t rescue the cell death induced by BFA, there observed higher levels of XIAP than BFA alone treated cells (Fig. 7e). Therefore the inhibition of caspase-3 activity rescues the target protein XIAP levels in cells pretreated with kaempferol.

## Discussion

Flavonoids are polyphenolic compounds often found in plant that contribute significantly in human diet especially via dietary sources like tea, wine, legumes, vegetables and fruits. Flavonoids have been consumed traditionally as folklore medicine. Previous reports suggest the diversified biological activities of flavonoids which include anti-inflammatory, anti-carcinogenic and anti-oxidative stress abilities. Kaempferol is an abundantly found flavonoid in edible and medicinal plants. It exhibits various pharmacological activities like antioxidant, anti-inflammatory, antimicrobial, anticancer, cardio-protective, neuroprotective, antidiabetic, anti-osteoporotic, estrogenic/anti-estrogenic, anxiolytic, analgesic and anti-allergic activities^28^.

The present study showed the ability of kaempferol, plant derived ER β agonist to inhibit ER stress induced cell death in cultured mammalian cells. This is consistent with the evidence that kaempferol inhibits the expression of certain key genes that regulate the UPR pathways. In this regard, we showed that kaempferol significantly decreased the BFA induced mRNA expression of UPR markers like GRP78 and CHOP in IMR32 and MDA-MB-468 cells. Furthermore, eIF2α and downstream elements of UPR pathway molecules like p38 MAPK have also been regulated by kaempferol. The stress kinase p38 MAPK and CHOP have been known to induce the expression of pro-apoptotic gene Bim and down-regulate the anti-apoptotic gene Bcl-2 under ER stress conditions. In our study, both BFA alone and kaempferol pretreated cells has decreased the mRNA expression level of Bcl-2 and Bim. The activation of caspase-3 was shown to degrade Bim in mouse osteoblast cells^56^. Therefore, the reduction in Bim expression level can be attributed to high caspase activation with BFA treatment in IMR32 cells.

Several studies have suggested that flavonoid compounds induce apoptosis of cancer cells and does not induce cell death of normal cells; in our study, kaempferol treatment did not change the activity of caspase 3/7 in neuroblastoma and in other cancer cells at 50 µM concentration; the effect of kaempferol in normal cells yet to be tested. Caspases are known to get activated during ER stress and they are essential for ER stress induced cell death. It has been shown that caspase 9 inhibitor repress ER stress induced cell death in primary neuronal cells following hypoxia/reperfusion induced ER stress^38,57^. A very recent study showed that ZVAD-FMK also inhibited ER stress. Furthermore, it has been demonstrated in the same study that ER stress downregulated many caspase substrates at the protein level; caspases influenced ER stress pathway both by directly cleaving its substrates and also by deactivating transcription factors essential for upregulation of genes involved in cell fate decisions^58^.

In our study, while investigating the specificity of the compound, we identified and realized that kaempferol inhibits cell death induced by multiple stimuli. Although kaempferol is not specific against ER stress induced cell death, it plays an interesting role of targeting caspases. Results of structural analysis using *in silico* approach as well as biochemical and kinetic studies using recombinant caspase enzyme strongly support the fact that a common allosteric mechanism is the base for caspase inhibition by kaempferol. Kaempferol is expected to bind to the dimerization interface and change the conformation of caspases and thereby affect the catalytic activity. However, this remains a hypothesis as we do not have direct evidence to support it by crystallographic study. Besides the demand for a high expense by its potency, the caspase inhibiting ability of this commonly occurring dietary flavonoid can be considered for therapeutic application. Furthermore, several reports have shown the association of ER stress and therapeutic opportunity of caspase inhibitors to neurodegenerative diseases. Currently, available inhibitors of caspases are mostly peptide based and are mainly used for research purpose; the synthetic peptide mimicking orthosteric inhibitors are shown to form toxic products during the course of metabolism *in vivo*^59^. In this study, the inhibition of caspase activation with ZVAD-FMK showed increased cell death in IMR32 cells induced with BFA (data not shown). Therefore, kaempferol may be evaluated further as ER stress/non-peptide caspase inhibitor.

Introducing caspase inhibitors particularly peptide based, as safe and effective therapeutics may face a lot of challenges considering multiple variables like specificity, selectivity, understanding the contribution of caspases in diseases, knowledge of therapeutic window and off target effects^60^. A major accomplishment is Emricasan a potent irreversible pan caspase inhibitor which is being evaluated in clinical trials for treatment of liver diseases. It has demonstrated significant tissue specificity with no effects on healthy individuals^61^.

Appreciable number peptidomimetics and small molecule inhibitors have been identified as either specific or pan caspase inhibitors^62^. Several published reports on caspase inhibition are either significant findings or in initial phase of development and their clinical efficacy is yet unexplored. In *in vitro* and *in vivo* models catechin derivatives were found to suppress apoptosis in hepatocytes by inhibiting caspase-3^63^.

Isatin analog compounds have inhibited caspase-3 effectively in *in vitro* enzyme assays using recombinant enzyme and in HeLa cells treated with cyclosporine^64^. Kaempferol demonstrated dose dependent inhibition of caspase 3/7 activity and exhibited anti-apoptotic against multiple cell death stimuli like BFA, tunicamycin, CDDO-Me, staurosporine, doxorubicin, TNFα and cycloheximide in various cell lines. Nevertheless, kaempferol may target other molecules as well. For instance, screening for DAPK1 specific inhibitors recently identified kaempferol as one of flavonoids interacting with DAPK1 for which efficacy in cell lines is yet unknown^65^. The loss and gain of function of DAPK1 is associated with various cancer and neurodegenerative diseases respectively^66^. Kaempferol has been shown to inhibit NADPH oxidase directly and thereby preventing PC12 cells from 4-hydroxynonenal (HNE) induced apoptosis^67^. Despite these previously known targets, we propose caspase inhibition as kaempferol’s mode of action that promotes survival in IMR32 cells because the contribution of these targets may not account for cell death induced by various stimuli we used in our study. Kaempferol inhibited ER stress and caspase 3 activation induced by ischemia reperfusion in cardiomyocytes and *ex vivo* model^68^. To the best of our knowledge, our study is the first report to demonstrate the activity of kaempferol against ER stress specific inducers and the mechanism of action of kaempferol in neuronal cells.

**Figure 8:**
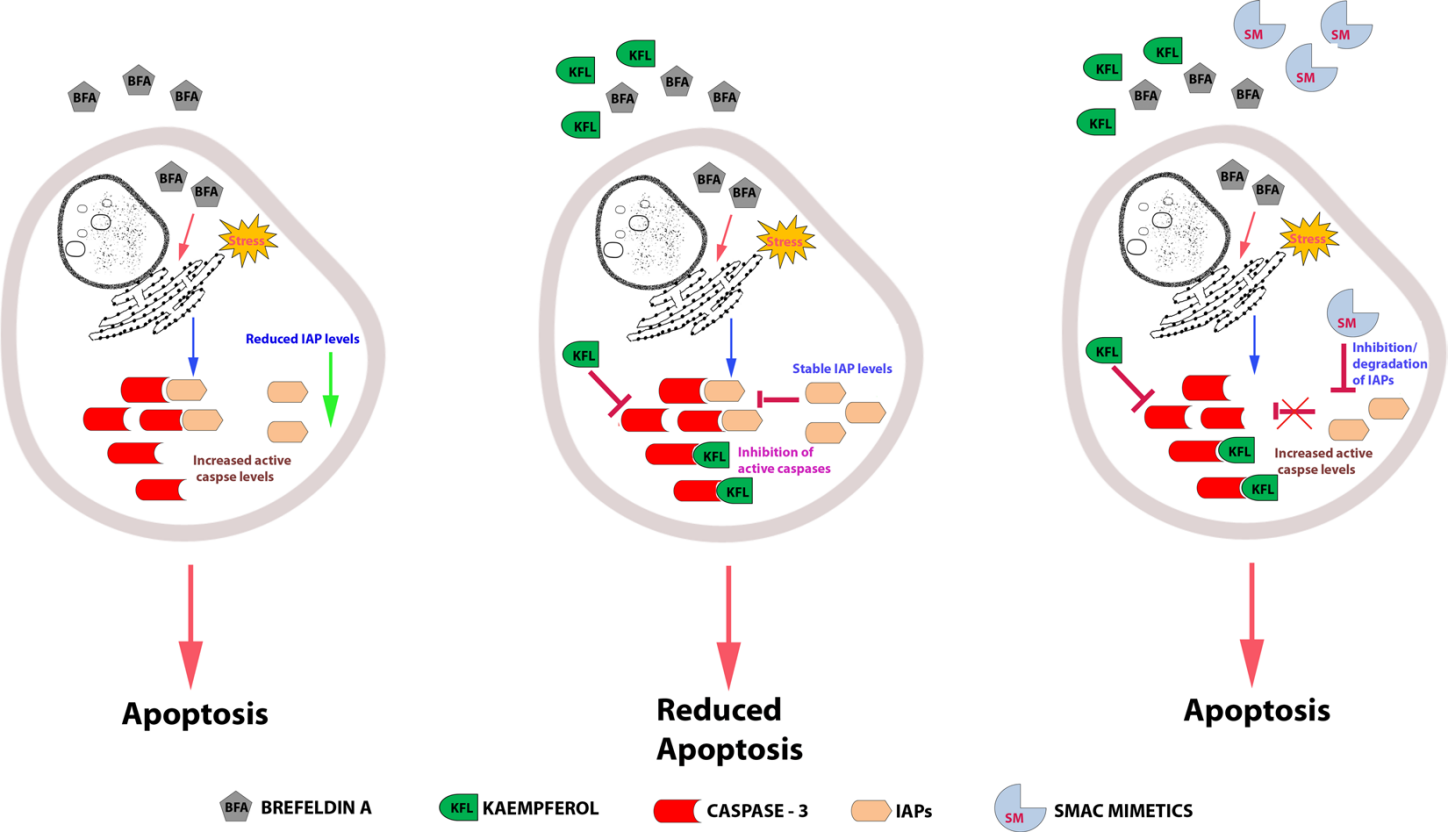
Graphical abstract. Kaempferol alleviates ER stress by inhibiting the executioner caspase 3/7. Endogenous inhibitor of apoptotic proteins-IAPs level did not get altered upon kaempferol treatment and the SMAC mimetics which are inhibitor of IAPs reverses the kaempferol mediated caspase inhibition and allows cell death to occur.

The catalytic activity of caspases is subject to inhibition by the IAPs. Eight distinct mammalian IAPs, including c-IAP1, c-IAP2 and XIAP have been identified; they target initiator caspase-9 and the effector caspase-3 and -7^69^, thereby protect cells from apoptosis. IAPs do not inhibit other caspases like caspase-8 and -6. In respect to ER stress, inducers such as BFA and tunicamycin induce PERK and activate IAPs to inhibit apoptosis in a PERK dependent manner^70^. In our study, BFA induced the expression of PERK and when pre-incubated with kaempferol it did not show alteration in the expression level. We observed a reduction in cellular caspase level by kaempferol treatment under ER stress condition. Also, we have shown that SMAC mimetics compounds were able to reverse the kaempferol mediated ER stress inhibition in IMR32 cells. Additionally, IAPs at protein level were observed to be at high level when compared to BFA alone treated cells. Altogether, we postulate the mechanism as illustrated in Fig. 8.

In conclusion, kaempferol can be developed into a therapeutic agent specifically for neurodegenerative diseases as there is increased ER stress, inflammation and oxidative stress causing disease driven loss of cells. It is worth to note that kaempferol has been shown to inhibit all these processes in various cell types. We present our preliminary finding on caspase inhibition by kaempferol; however, further crystallography validation is a prime requisite. After which, kaempferol could be an ideal candidate to investigate the pharmacological aspect in *in vivo* disease conditions wherein caspase dependent cell death is involved.

## Materials and Methods

### Reagents

Brefeldin A, Tunicamycin, Q-RT PC and Dimethyl sulphoxide (DMSO) were purchased from Sigma Aldrich. DMEM, Fetal Bovine Serum, Antibiotic and Antimycotic solution, MTT and HiPurA RNA isolation kit were obtained from HiMedia, India. Kaempferol was purchased from Natural Remedies Pvt. Ltd., Bangalore. DPN (2,3-Bis(4-hydroxyphenyl)-propionitrile), PHTPP were purchased from Alfa Aesar (Hyderabad, India) and Cayman Chemicals, USA respectively. GDC0152, LC161 and Z-VAD-FMK from MedChem express, USA. CellTiter – Glo Luminescent cell viability assay kit, Caspase - Glo 3/7 assay kit, cDNA conversion kit was obtained from Applied Biosystems, India. SYBR Premix Ex Taq kit was purchased from TaKaRa Bio-Inc, India. Antibodies were purchased from Cell Signaling Technology, Danvers, MA, USA and Novus Biologicals, USA. For MTT assays, polystyrene, flat bottom and clear wells plates were used while for luminescence based assays solid white plates were used.

### Cell culture conditions

Human IMR32, MDAMB 468, HeLa, MCF7 and mouse Neuro2A cells obtained from NCCS, Pune, India were cultured in DMEM supplemented with 10% Fetal Bovine Serum and maintained at 37 °C in an incubator with humidified atmosphere containing 5% CO_2_ and 95% air. Antibiotic and Antimycotic solution was added to prevent microbial growth in the cell culture.

### Cytotoxicity assays

MTT and CellTiter-Glo luminescent cell viability assays were performed to estimate the cell viability. For MTT assay, cells were plated at density of 5 × 10^3^ cells/well in 96 well plates on day 0. Next day, medium was removed from each well and then pre-treated with Kaempferol or other selected compounds for 90 minutes followed by treating the cells with ER stress inducers. After 24 h of incubation, 20 µl of MTT (20 mg/ml) was added to each well and then incubated back for 3 hours at 37 °C in an incubator with humidified atmosphere containing 5% CO_2_ and 95% air. The formazan product which was formed in the cell was dissolved with 100% spectroscopic grade DMSO. ELISA reader (BioRad) was used to read the absorbance at 570 nm. For CellTiter-Glo assay, same procedure was followed in a 96 wells plate (polystyrene, flat bottom and white plate) except of adding MTT. To this, CellTiter-Glo assay solution was added. After 5 minutes of incubation, luminescence was measured using Luminometer (Berthold, Germany).

Alternatively cell viability was assessed with trypan blue dye exclusion assay. 5 × 10^4^ cells were seeded in a 24 well plate and treated with Brefeldin A or in combination with Kaempferol in triplicate. After 24 hour of incubation the cells were collected from media along with trypsinized cells and counted for blue stained cells for dead and transparent for live cells after 2 minute incubation with 0.4% trypan blue (w/v) dissolved in PBS.

### Caspase 3/7 activity assay

Cells were plated at density of 8 × 10^3^ cells/well in a 96 well plate. Cells were incubated for overnight and pre-treatment with test compound, followed by treatment with ER stress inducers for specified hours. Caspase Glo- 3/7 solution was added and incubated at 22 °C for 30 minutes as specified by the manufacturer. Luminometer (Berthold) was used to measure the luminescence.

### RNA extraction and Quantitative RT-PCR

Total RNA was extracted using HiPurA^Tm^ Total RNA Miniprep purification kit from Himedia laboratories. Subsequently, 2 µg of total RNA was converted into cDNA using Reverse Transcriptase and manufacturer’s instructions were followed. For QPCR (AB 7500, Step One), 2 µl of the RT product was used with gene specific primers in conjunction with SYBR green. Human18s rRNA was used as internal control and relative gene expression was calculated using 2^−ΔΔ***CT***^ *method*. *List of primers used in this study is tabulated in* Supplementary Table 2.

### Immunoblotting

Immunoblotting was carried out as described earlier by *Ravanan et al*.^71^. In brief, cells were plated in 60 mm dishes at the density of 6 × 10^5^ cells/dish. After overnight incubation, cells were pre-treated with kaempferol for 90 min and then treated with BFA for various time points. Cells were, washed with ice cold PBS and lysed with RIPA buffer containing 1X Protease cocktail and 1X PhosSTOP. Total protein concentrations were quantified by Folin’s assay. Immunoblotting analysis was done by loading 50 µg of protein in each lane for SDS-PAGE. Antibodies used in this study were obtained from Cell Signalling Technology (Beta actin#8457, eIF2α#, Phospho-eIF2α#3597, p38 MAPK#8690 and Phospho-p38 MAPK#4511) and ABclonal (GRP78 #A0241). Densitometry analysis of developed blots were carried out using NIH-ImageJ software and normalized with respect to β-actin as internal control.

### Immunocytochemistry and Microscopy

Immunocytochemistry was carried out as described in the Cell signaling Technology (CST)guide. Briefly, 5 × 10^4^ cells/wellwere seeded in a 24 well plate. After overnight incubation, cells were treated with the compounds for indicated time. The cellswere then washed with 1XPBS and fixed with 4% Formaldehyde for 15 min. The cells were washed again with 1XPBS and incubated with 0.5% skimmed milk containing 0.3% tritonX100 for blocking nonspecific binding, for 1 hour. Immunostaining was done using cIAP1 (#NBP2-27190SS) and cIAP2 (#NB100-1666), XIAP (#NB100-56183) specific antibodies (Novus biologicals, USA) and Anti-rabbit Alexa Flour 594 (#8889, Cell Signaling Technologies, USA) as a secondary antibody. Phase contrast and fluorescence images of cells under treated and non-treated conditions were captured using EVOS FLoid imaging station (Thermo Fisher, USA) with 20X fluorite objective and LED light cubes containing hard coated filters (blue and red). For a particular protein expression study, imaging parameters were kept constant throughout the imaging no image modifications were done post imaging.

### Recombinant enzyme activity assay

For enzyme inhibition assays, recombinant Caspase 3 enzyme was obtained from Enzo Life sciences, BIOMOL Cat. #SE-169 and Caspase 3/7 glo assay reagent from Promega. 2 units of enzyme was used per well in a reaction mixture formed with 10 mM HEPES and 0.1% BSA. Reduction in the amount of luminescence would reveal the extent of inhibition caused by the test compounds from cleaving the substrate Z-DEVD-amino luciferin by human recombinant caspase-3 enzyme. Non liner regression analysis for Michelis-Menton kinetics and Curve fit for sigmoidal dose response (variable slope) was done using GraphPad Prism 5.0.

### *In silico* analysis

Docking studies were carried out using AutoDock Suite 4.0. In brief, X-ray crystallographic structure of caspase-3 (PDB ID: 3DEJ), caspase-7 (PDB ID: 4FEA) and caspase-9 (PDB ID: 1NW9) were used as the target receptor. Ligands were retrieved from PubChem database in their 3D conformer form and corrected for protonation. Non- polar hydrogens were assigned for macromolecule. Gasteiger charges were set for the ligands and Kollman charges were added for the receptor using AutoDock Tools 1.5.6. The macromolecule was kept rigid during docking study. Rotatable bonds for ligand molecules were assigned. A grid centre was made on centre of macromolecule covering the entire protein surface, which functioned as the search space. AutoGrid 4.0 was carried out to generate the map files for the flexible atoms and Lamarckian Genetic algorithm was used to define the docking parameter file (DPF) for AutoDock 4.0. Validation for docking procedure was done by re-docking the ligand 0TE with the active caspase 7 structure solved by X-ray crystallography. The re-docked orientation was compared to the crystal structure of docked complex (PDB ID: 4FEA). Based on the non-bonded interactions, H-bonds, torsional and desolvation energies, binding energy of the complexes were calculated for the confirmation with best binding pose and minimum energy. The binding interactions were studied using LigPlot^+^ v. 1.4 for 2D interactions. PyMol V. 1.7.4 and UCSF Chimera 1.10.2 were used for studying 3D interactions.

### Statistical analysis

All data are presented as the Standard error of mean (S.E.M) of at least two independent experiments. Statistical comparisons were performed using one-way analysis of variance (ANOVA) followed by Bonferroni’s multiple comparison test (Graphpad Prism, version 5.0) and p-value of less than 0.05 was considered statistically significant.

## Electronic supplementary material

Supplementary Information
