# Supplementary material for: Kaempferol mitigates Endoplasmic Reticulum Stress Induced Cell Death by targeting caspase 3/7
Source: Sci Rep. 2018 Feb 1;8:2189. doi: 10.1038/s41598-018-20499-7 (PMC5794799; doi:10.1038/s41598-018-20499-7)
Supplement: Supplementary file 1 — Supplementary Information [file 41598_2018_20499_MOESM1_ESM.pdf]

Kaempferol mitigates Endoplasmic Reticulum Stress Induced Cell Death by targeting caspase 3/7

Ahmad Abdullah and Palaniyandi Ravanan*

Apoptosis and Cell Survival Research Lab, Department of Biosciences, School of Biosciences and Technology, VIT University, Vellore.

*For Correspondence: Dr. P. Ravanan, Associate Professor and Head of the Department of Biosciences, School of Biosciences and Technology, VIT University, Vellore, Tamil Nadu, INDIA - 632014. Email: ravanan.p@vit.ac.in; Fax: 91-416-2243092

Supplementary Figure 1:

a.

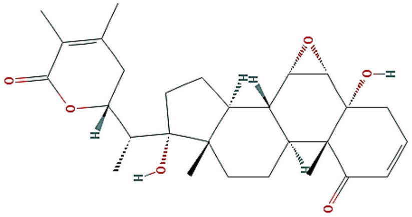

Withanone

b.

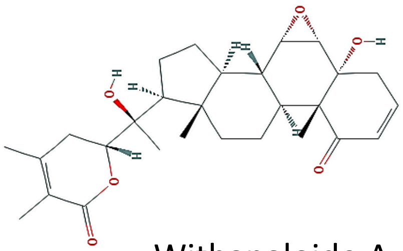

Withanaloide A

c.

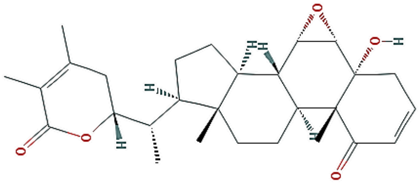

Withanaloide B

d.

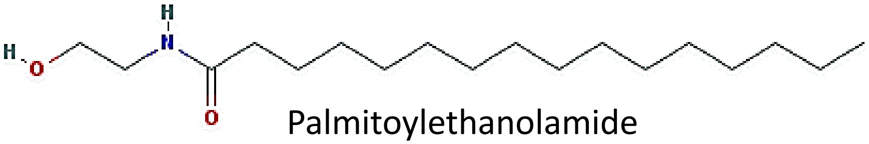

Palmitoylethanolamide

e.

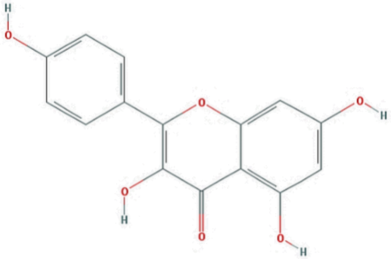

Kaempferol

f.

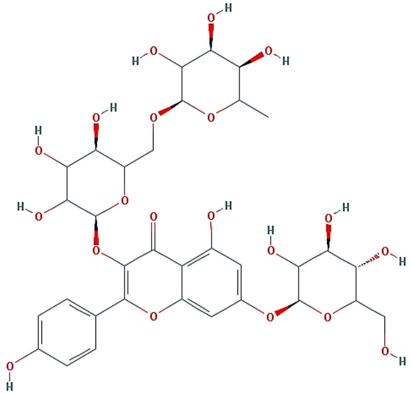

Kaempferol 3-O-Robinobioside -7-O-Glucoside

g.

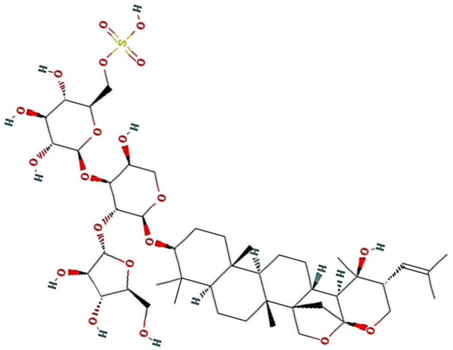

Bacopaside I

h.

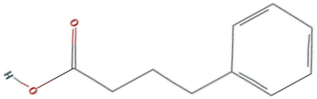

4- Phenyl butyric acid

i.

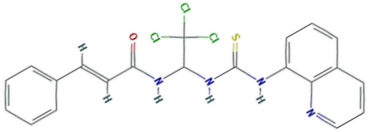

Salubrinal

Supplementary Figure 2:

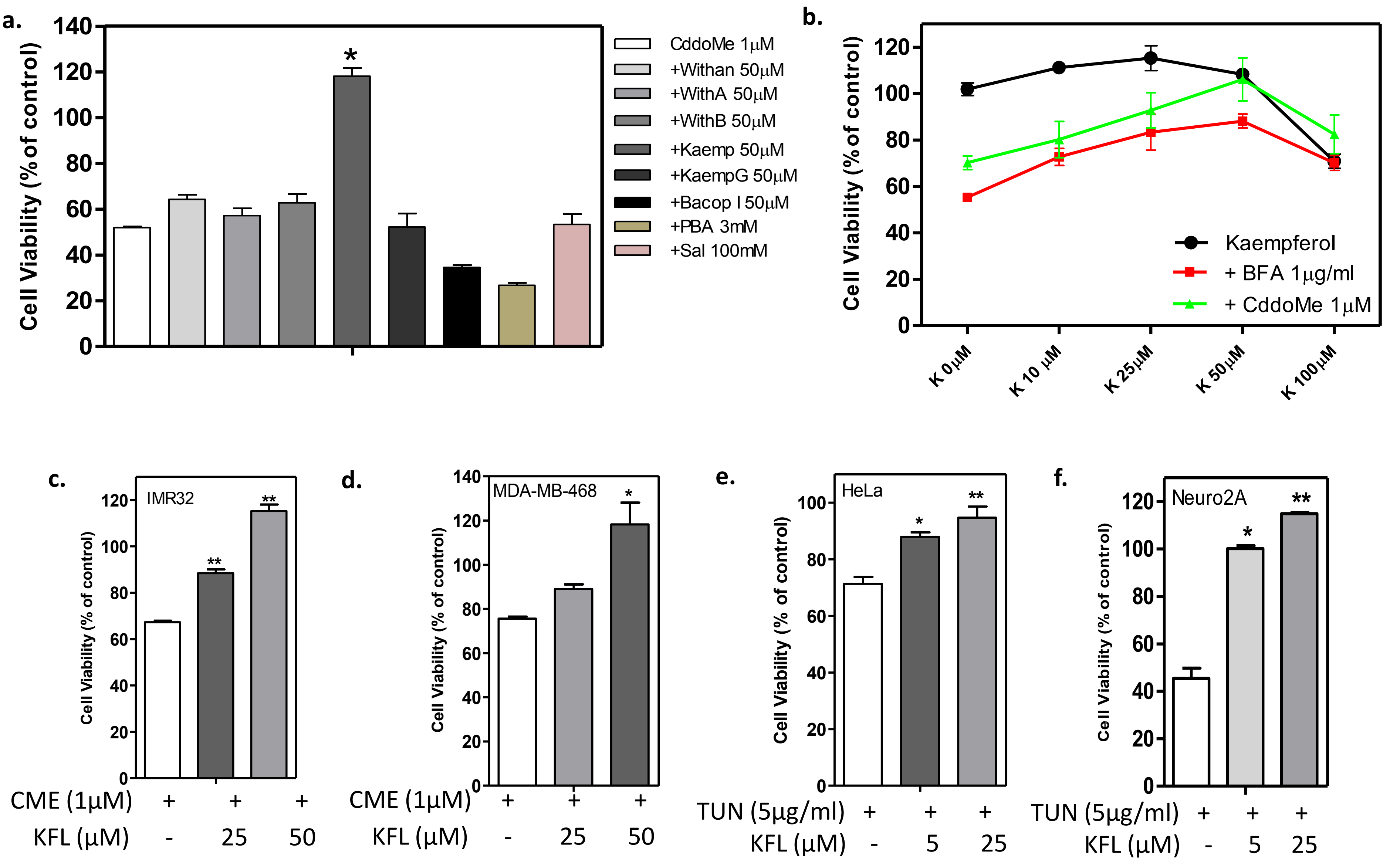

Supplementary Figure 3:

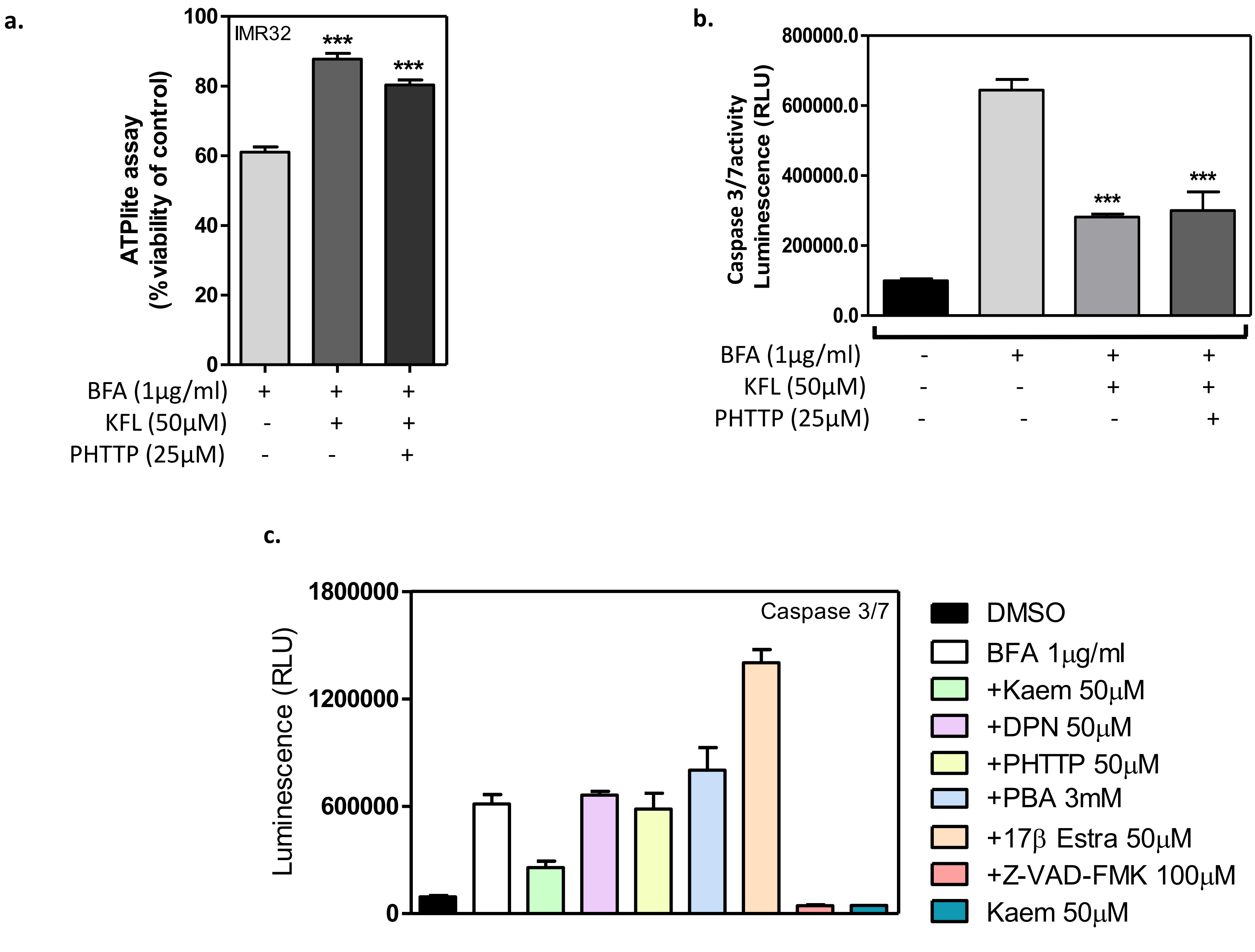

Supplementary Figure 4:

a.

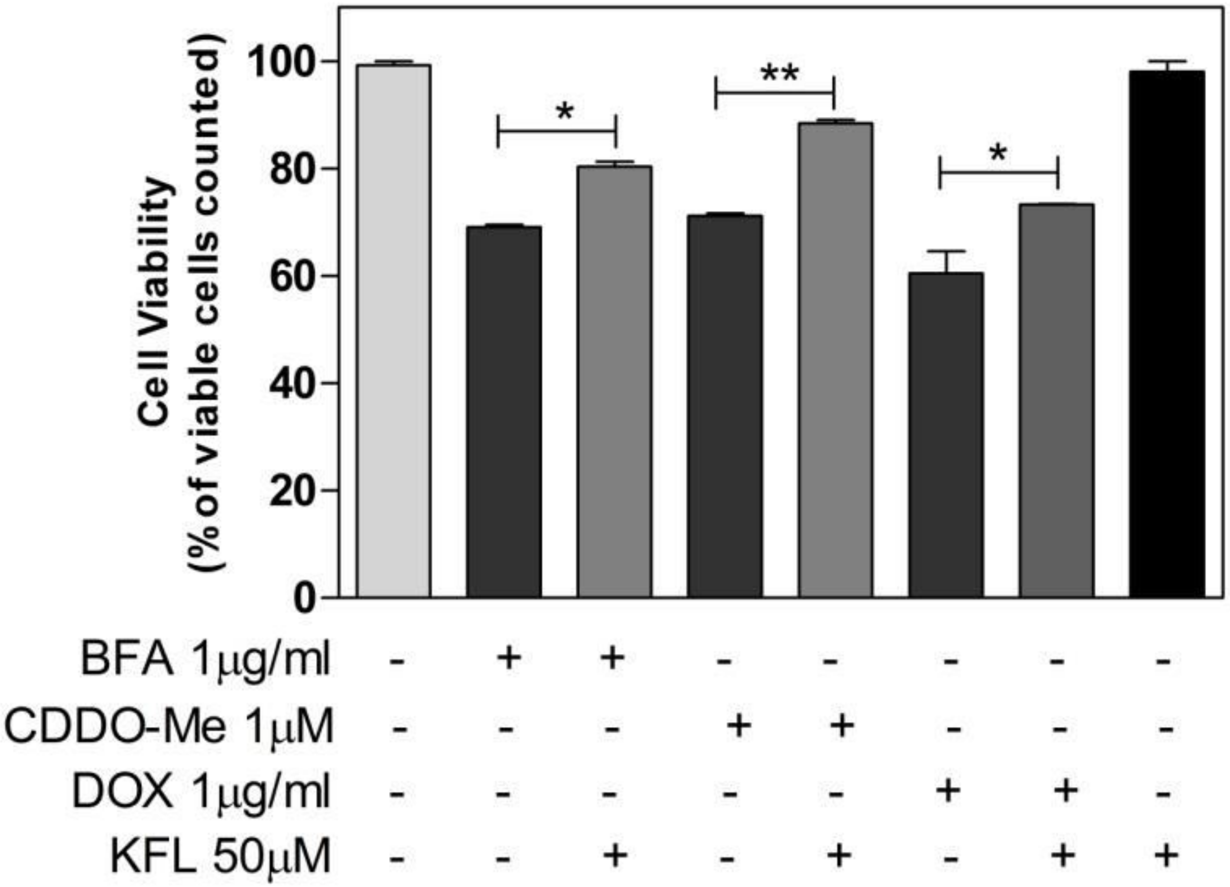

b.

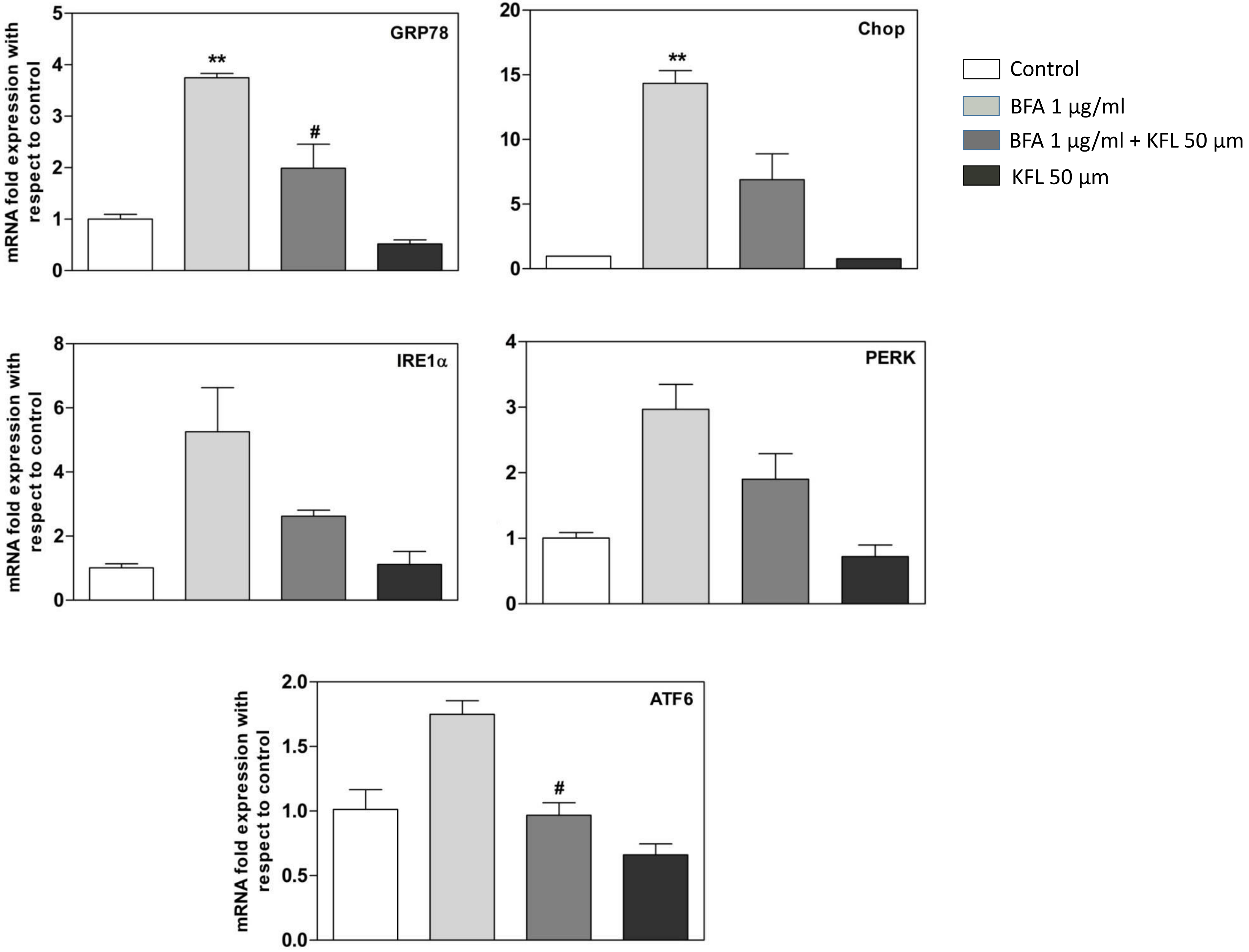

GRP78
Chop
IRE1α
PERK
ATF6
mRNA fold expression with respect to control
**
#
Control
BFA 1 µg/ml
BFA 1 µg/ml + KFL 50 µm
KFL 50 µm

c.

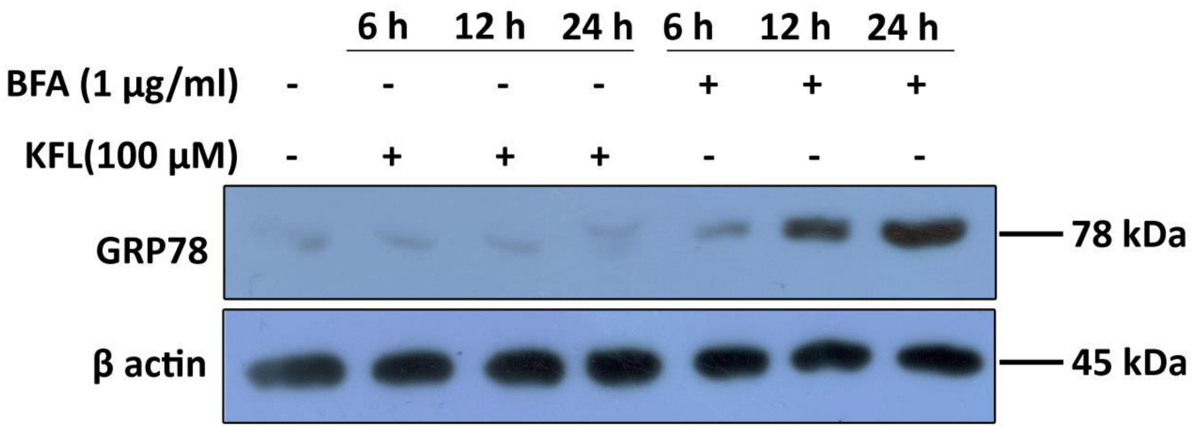
6 h
12 h
24 h
6 h
12 h
24 h
BFA (1 μg/ml)
- - - - + + +
KFL(100 μM)
- + + + - - -
GRP78
β actin
78 kDa
45 kDa

d.

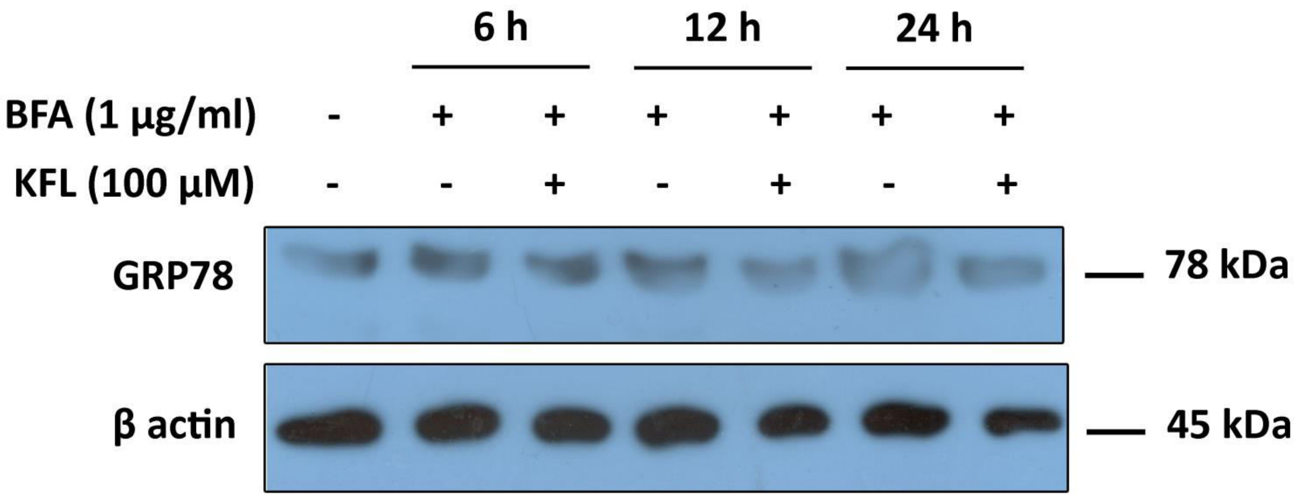
6 h
12 h
24 h
BFA (1 μg/ml)
- + + + + + +
KFL (100 μM)
- - + - + - +
GRP78
β actin
78 kDa
45 kDa

Supplementary Figure 5:

a.

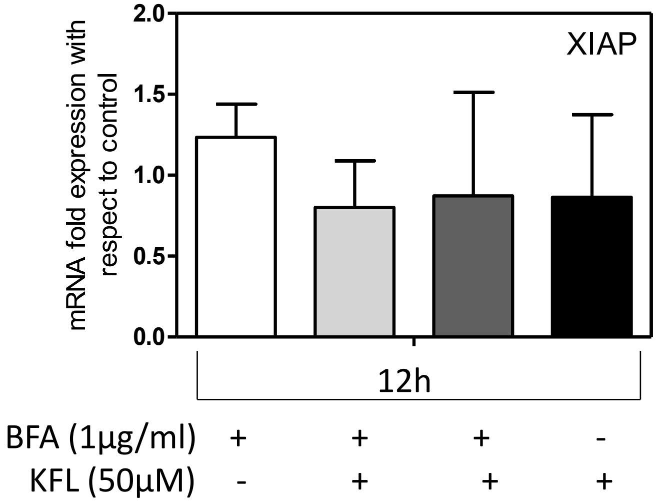

b.

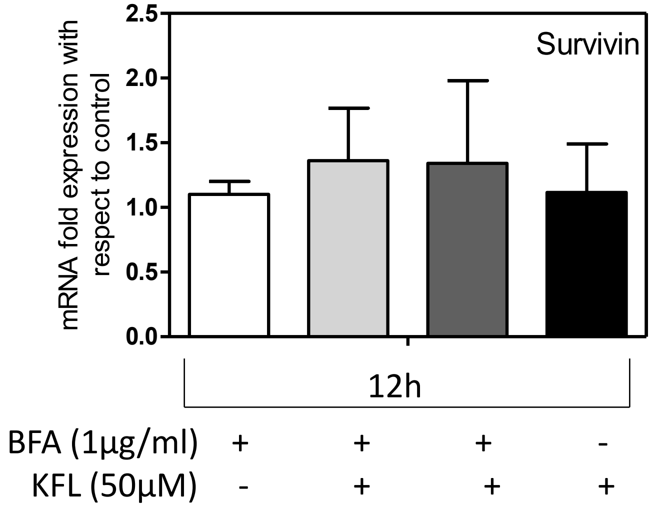

Supplementary Figure 6:

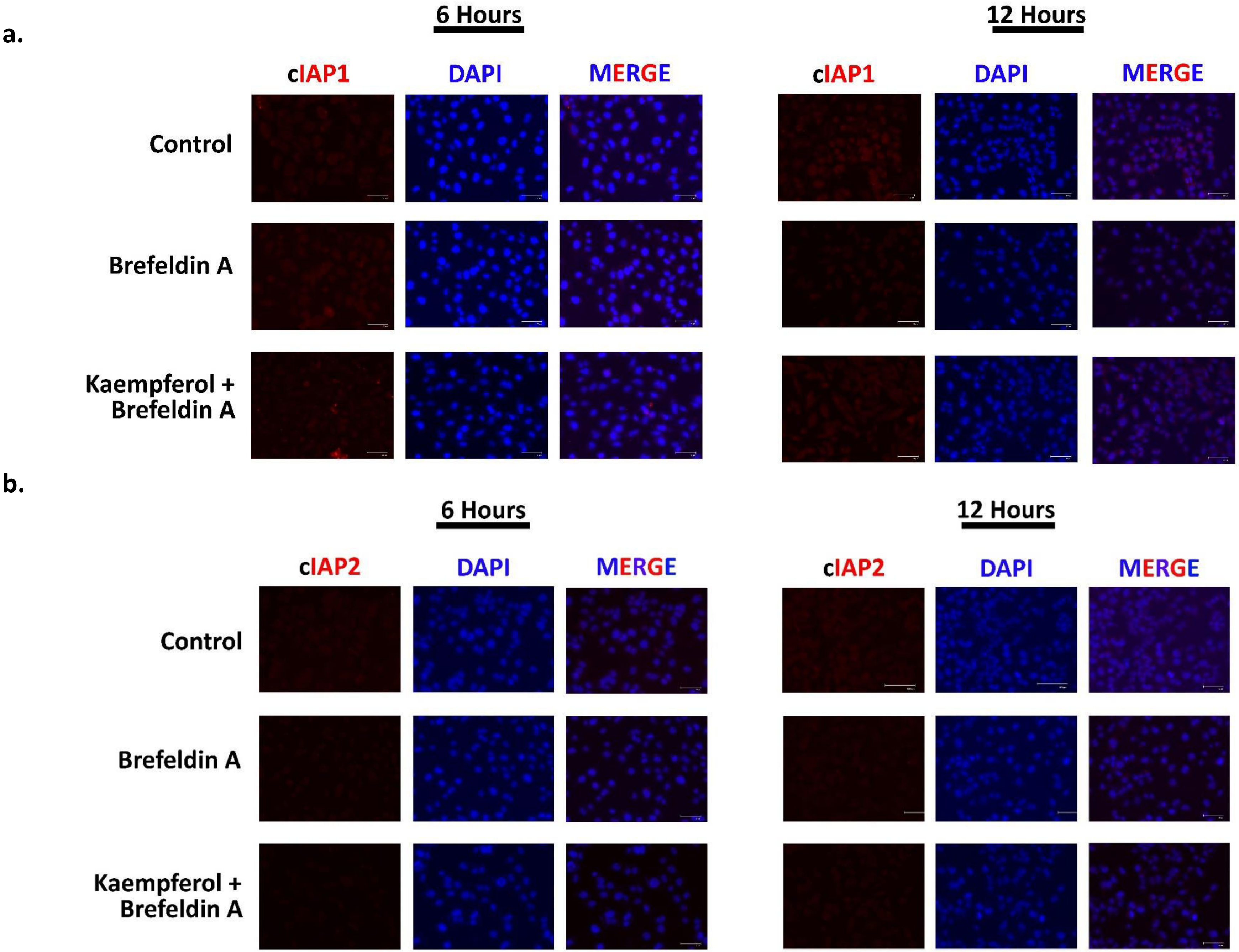

Supplementary Figure 7:

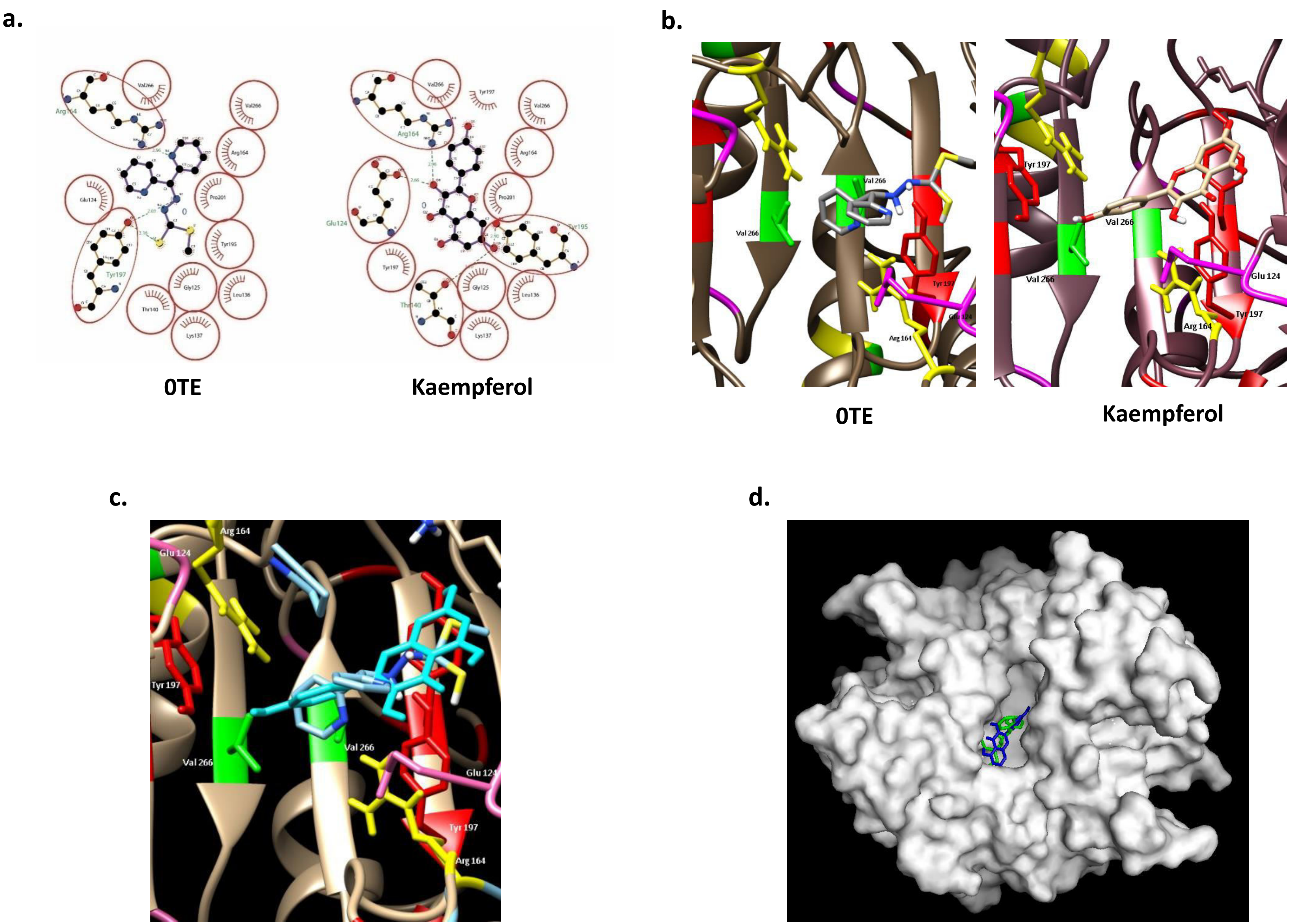

Supplementary Figure 8:

a.

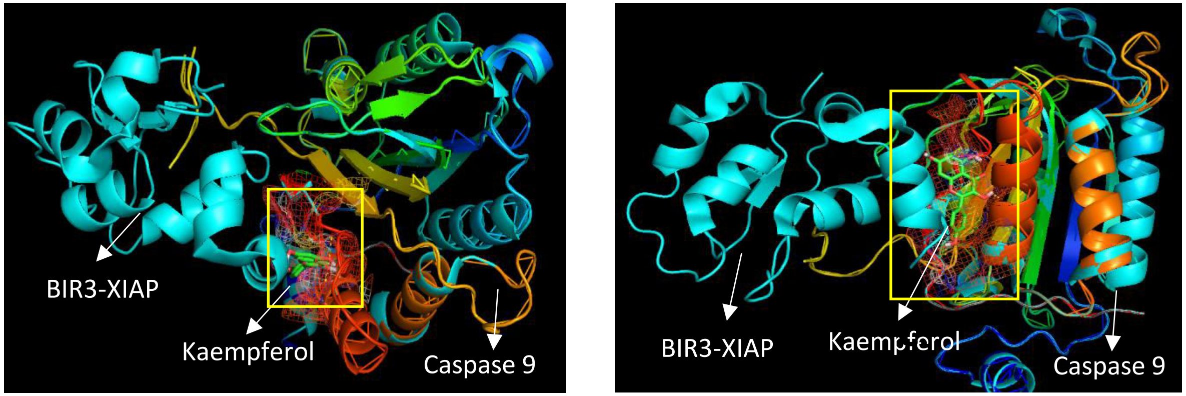

b.

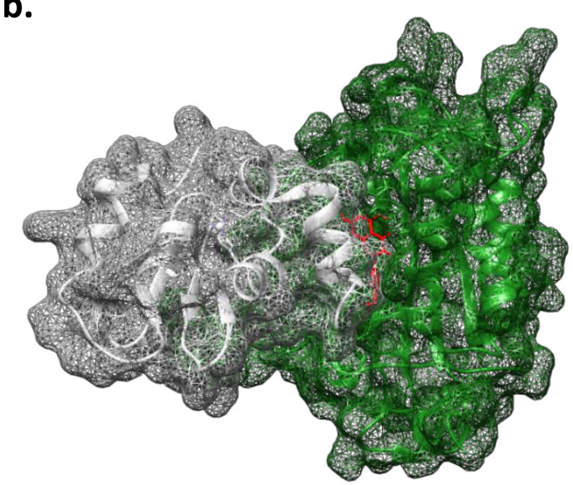

c.

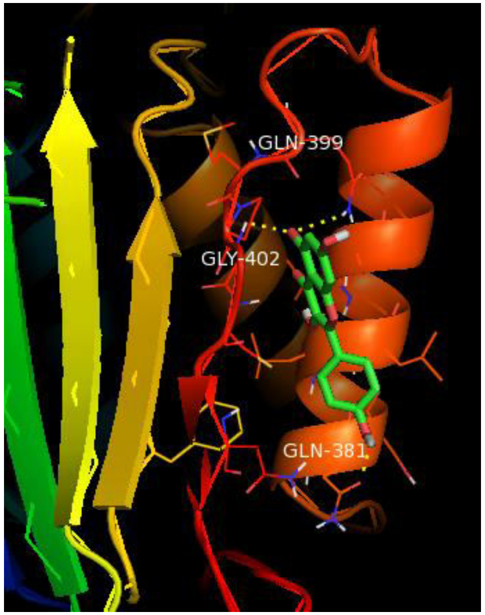

d.

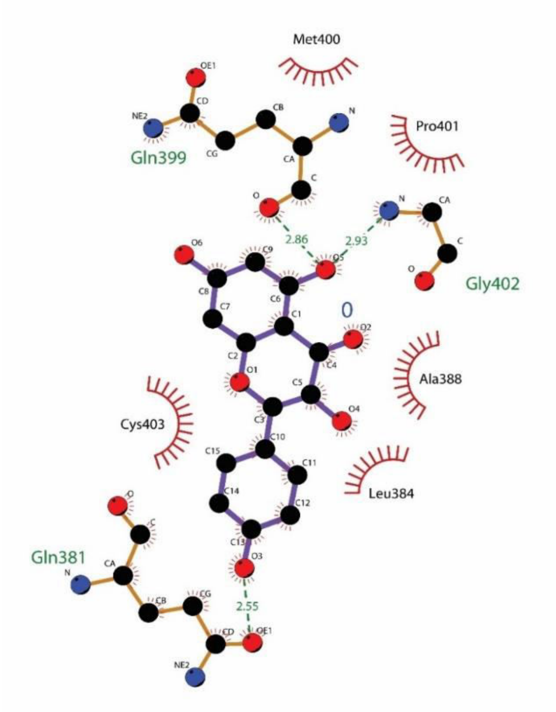

Supplementary Figure 9:

a.

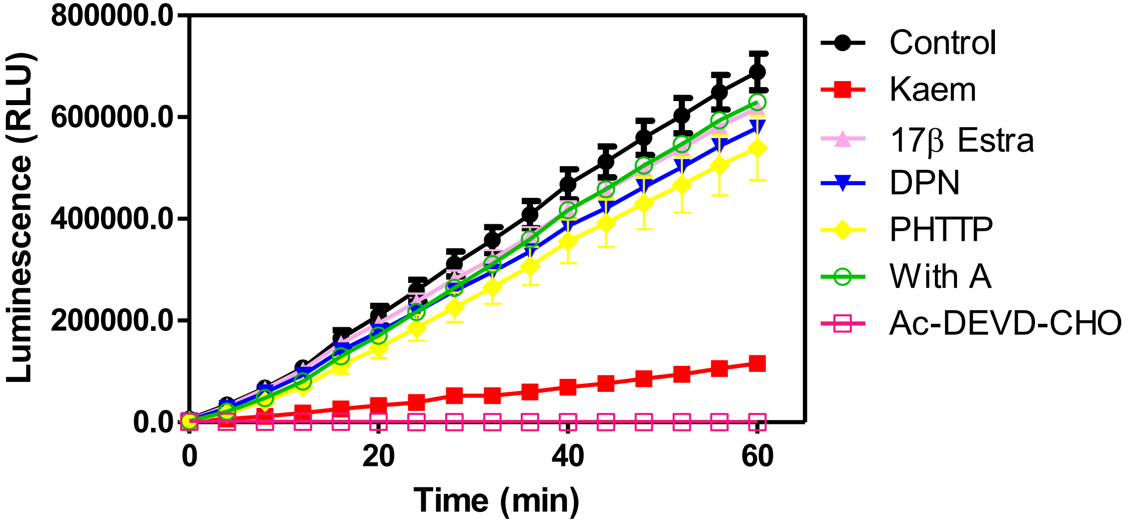

b.

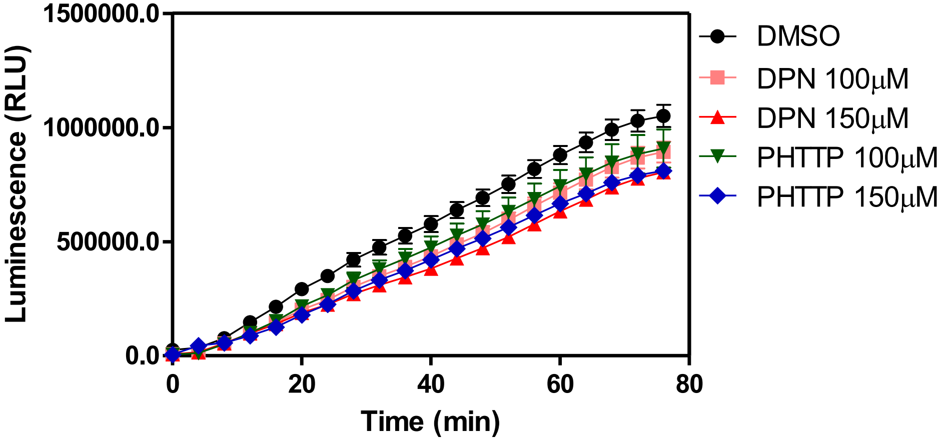

c.

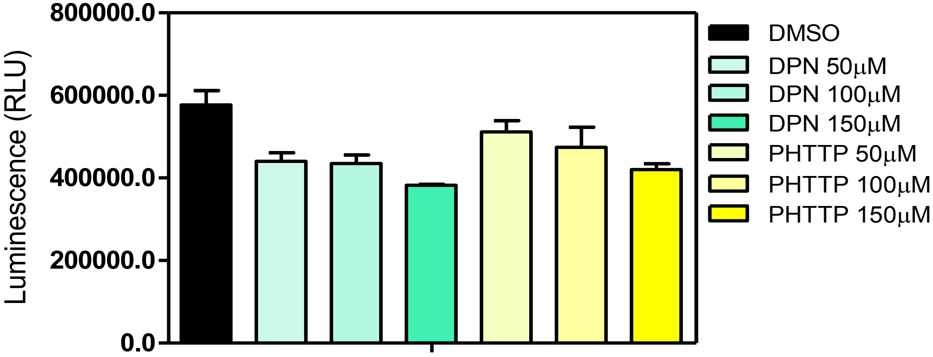

d.

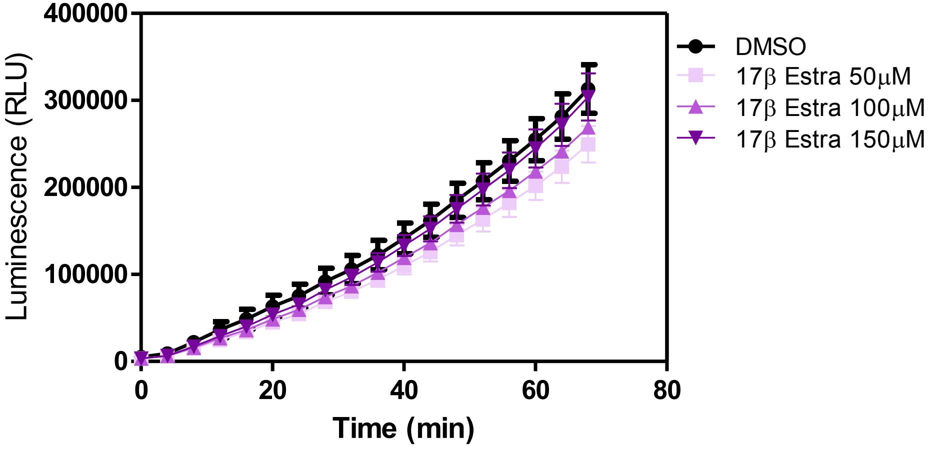

400000
300000
200000
100000
0
Luminescence (RLU)
0
20
40
60
80
Time (min)
DMSO
17β Estra 50μM
17β Estra 100μM
17β Estra 150μM

e.

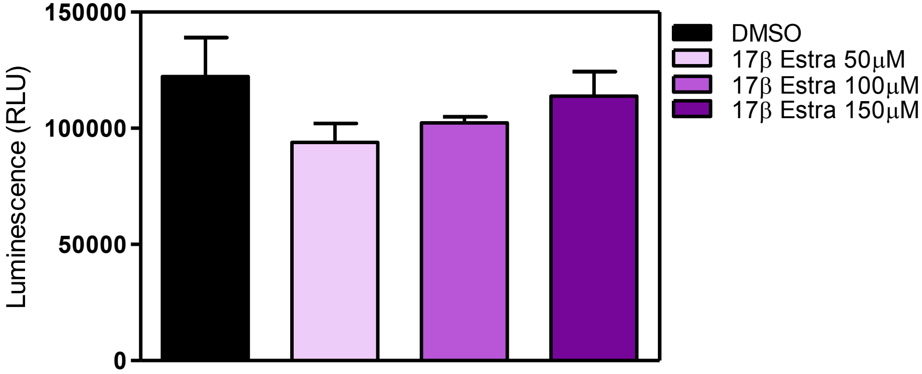

150000
100000
50000
0
Luminescence (RLU)
DMSO
17β Estra 50μM
17β Estra 100μM
17β Estra 150μM

Supplementary table 1. Docking scores obtained for Estrogen receptor modulators using Autodock 4.0

	Binding energy ΔG (kcal/mol)				
	0TE	Kaempferol	DPN	PHTTP	17β Estradiol
Caspase 3	- 4.75	- 6.58	- 5.46	- 4.82	--------
Caspase 7	- 2.83	- 4.01	- 2.94	- 3.74	--------
Caspase 9	- 4.15	- 6.01	- 6.65	- 3.59	- 4.02

S. Table 1. Docking scores obtained for estrogen receptor modulators compared to the known caspase inhibitor 0TE. 17β estradiol showed non favorable interactions with caspase 3 and 7 .

Supplementary table 2. List of primers used in this study for Q-RT-PCR.

Gene	Sense (5'-3')	Anti-Sense (5'-3')
**18s**	GCCGCTAGAGGTGAAATTCT	CATTCTTGGCAAATGCTTTC
**Grp78**	CAACCAACTGTTACAATCAAGGTC	CAAAGGTGACTTCAATCTGTGG
**CHOP**	CAGAGCTGGAACCTGAGGAG	TGGATCAGTCTGGAAAAGCA
**ATF6**	TTGGCATTTATAATACTGAACTATGGA	TTTGATTTGCAGGGCTCAC
**IRE1 alpha**	CCATCGAGCTGTGTGCAG	TGTTGAGGGAGTGGAGGTG
**PERK**	CAGTGGGATTTGGATGTGG	GGAATGATCATCTTATTCCCAAA
**DR5**	AGGTGAAGTGGAGCTAAGTC	TCACTCCAGGGTGTACAATC
**BIM**	TGGCAAAGCAACCTTCTGATG	GCAGGCTGCAATTGTCTACCT
**Bcl2**	CTGCACCTGACGCCCTTCACC	CACATGACCCCACCGAACTCAAAGA
**cIAP1**	AGCTAGTCTGGGATCCACCTC	GGGGTTAGTCCTCGATGAAG
**cIAP2**	TGGAAGCTACCTCTCAGCCTAC	GGAACTTCTCATCAAGGCAGA
**XIAP**	TTTGCCTTAGACAGGCCATC	TTTCCACCACAACAAAAGCA
**SURVIVIN**	TCCACTGCCCCACTGAGAAC	TGGCTCCCAGCCTTCCA

Figure legends: (Supplementary)

Supplementary Figure 1. **Structural representation of the compounds retrieved in 2D confirmation from Pubchem used for screening in this study.**

Supplementary Figure 2. **Cyto-protective action of kaempferol against different ER stress inducers in multiple cell lines.**

IMR32 cells pretreated with 50μM of phyto-compounds for 90 minutes and ER stress was induced by adding 1μM CDDO-Me for 24 hours. Cell survivability was analyzed using MTT assay. **(a)**.(Withan- Withanone; With A- Withanaloide A; With B-Withanaloide B; kaemp- Kaempferol; KaempG- Kaempfeol-3-O-robinbioside-7-O-glucoside; BacopI- Bacopaside I; PEA- Palmitoylethanolamide; PBA- Phenyl Butyric acid; Sal- Salubrinal).

Dose response curve showing the viability of IMR32 cells in kaempferol treatment alone or in combination with ER stress inducers Brefeldin A (1 μg/ml) and CddoMe (1 μM) for 24 hours. MTT assay was performed for determining cell viability. Data represented as average ±SEM of three independent experiments performed in triplicate. **(b)**

After the pre-treatment with kaempferol for 90 minutes IMR32, MDA-MB-468 cell lines **(c, d)** and Neuro2A, Hela cell lines **(e,f)** were treated with CDDO-Me (1 μM) and Tunicamycin (5 μg/ml) respectively for 24 hours. Data represented as average ±SEM; n=2.* represents the significance between cell death inducer alone treated condition compared to kaempferol pre-treated condition, at $p \leq 0.05$ (one way ANOVA).

Supplementary Figure 3. **Cyto-protective effect of estrogen receptor modulators.**

IMR32 cells pretreated with kaempferol 50 μM in presence and absence of PHTTP 25 μM for 90 minutes. ER stress was induced with 1 μg/ml of Brefeldin A. After 24 hours of treatment, cellular ATP levels and Caspase 3/7 activity were measured using CellTitreGloassay and caspase 3/7 Glo assay respectively. Experiments were performed in triplicate (n=3) and data represented as average of ±SEM **(a, b)**.* represents the significance between cell death inducer alone treated condition compared to kaempferol pre-treated condition, at $p \leq 0.05$ (one way ANOVA).

Active caspase inhibition by kaempferol pretreatment compared to other ER modulators in *in vitro* model using IMR32 cells. ER stress was induced with Brefeldin A (1 μg/ml) for 24 hours. Data represented as average of ±SEM of experiment performed in duplicate (n=2) **(c)**.

Supplementary Figure 4. **Cyto-protective effect of kaempferol under apoptotic stimuli.**

Viable cell count assessment performed after 24 hours of exposure to cell death inducers using Trypan blue exclusion assay in IMR32 cells. Data represented as average of ±SEM of six counting for each condition, from two independent experiments performed in triplicate **(a).**

ER stress markers mRNA expression at 24 hours after the induction of stress. *represents the significant increase in expression of BFA treated cells with respect to control cells; #represents the reduction in gene expression in kaempferol pre-treated cells when compared to BFA alone treated cells ($p \leq 0.05$; one way ANOVA). **(b)**.

Expression of ER stress marker protein GRP78 in IMR32 cells in absence and in presence of Brefeldin A upon treatment with higher concentration of kaempferol (100 μM) **(c, d).**

Supplementary Figure 5. **Transcriptional regulation of anti-apoptotic proteins XIAP and SURVIVIN.**

IMR32 cells pre-treated with kaempferol for 90 minutes and ER stress was induced with BrefeldinA(1 μg/ml) for 12 hours. With the pre-treatment of kaempferol no significant regulation of XIAP **(a)** and Survivin**(b)** was seen against Brefeldin A induced ER stress. Data represents the average ±SEM of two independent experiments performed in duplicate.

Supplementary Figure 6. **Immunocytochemistry analysis of cIAP1 and cIAP2 protein expression.**

Images representing the expression of cIAP1**(a)** and cIAP2**(b)** proteins in IMR32 cells upon induction of ER stress with Brefeldin A (1 μg/ml) for 6 hours and 12 hours with/without pretreatment with kaempferol (50 μM).

Supplementary Figure 7. ***In silico* analysis of kaempferol binding to caspase 3 enzyme.**

Docking studies on binding of 0TE and Kaempferol to caspase 3 dimer interface using PDB:ID 3DEJ as protein structure. Hydrophobic interactions and hydrogen bond formation were analyzed using LigPlot$^+$ 2D interaction diagrams (**a**). 3D Binding site analysis was performed using UCSF chimera and PYMOL (**b&c**). Surface diagram showing the binding pocket of 0TE and kaempferol at the same allosteric site in the dimerization region of caspase 3 dimer (**d**).

Supplementary Figure 8. **Analysis of kaempferol bound caspase 9 monomer docked structure.**

Interaction studies on kaempferol binding with caspase 9 monomer (PDB:ID 1NW9).Top and side view of superimposed structures of kaempferol and XIAP-BIR3 with caspase 9 monomer, showing same site of interaction (**a**). Meshed surface showing the binding pocket for kaempferol in the BIR3 interaction site of caspase 9 monomer (XIAP-BIR3 domain- **Grey**; Caspase 9 monomer- **Green** and Kaempferol- **Red**) (**b**).3D

docked pose of kaempferol showing interactions with caspase 9 monomer (**c**). 2D LigPlot+ interaction analysis of kaempferol with the dimerization site amino acid residues of caspase 9 monomer (**d**).

Supplementary Figure 9. **Inhibitory effect of Estrogen Receptor modulators on human recombinant caspase 3 enzyme.**

Inhibition of caspase 3 activity on cleavage of Z-DEVD-amino luciferin substrate by estrogen receptor modulators (Kaempferol, DPN. PHTTP and 17β estradiol) with increase in time; Withanaloide A as (-)control and Ac-DEVD-CHO as (+)control for inhibition activity (**a**).Inhibition of human recombinant caspase 3 enzyme activity by DPN, PHTTP and 17β estradiolat various concentrations, 50, 100 and 150 µM analyzed using Caspase 3/7 glo assay (**b,c,d and e**).Data represented as average of ±SEM of experiment performed in duplicate (n=2).
